# In Silico Prediction of Chronic Oral Reference Doses for PIANO Target Analytes

**DOI:** 10.3390/toxics14060529

**Published:** 2026-06-18

**Authors:** Paul D. Rockswold, Gregory J. Joseph, Elaine A. Merrill, Christopher S. Waldron, James S. Smith

**Affiliations:** 1 Defense Centers for Public Health, Portsmouth, VA 23708, USA; 2 Battelle Memorial Institute, Columbus, OH 43201, USA; 3 Navy and Marine Corps Force Health Protection Command, Portsmouth, VA 23708, USA; 4 Pioneer Technologies Corporation, Olympia, WA 98503, USA

**Keywords:** chronic oral reference doses (RfDs), quantitative structure–activity relationship (QSAR) models, molecular descriptors, hydrocarbons, petroleum compounds, stepwise multivariable linear regression

## Abstract

Characterizing the human health risk posed by constituents in drinking water is often challenging due to a lack of published toxicity values. The PIANO (Paraffin, Isoparaffin, Aromatic, Naphthene, and Olefin) analytical method measures nearly 300 compounds in JP-5 jet fuel, 43 of which have published oral reference doses (RfDs). The remaining compounds are typically assigned surrogate toxicity values. We predict RfDs for 290 PIANO compounds using Quantitative Structure–Activity Relationship (QSAR) models based on stepwise linear regression of 2-dimensional molecular descriptors (MDs) and published toxicity values. Five training groups, created by randomly selecting 80% of the non-PIANO compounds and 50% of the 43 PIANO compounds that have RfDs within a master dataset of 1113 compounds, were used to develop five QSAR models. We used the geometric means of four QSAR model results of sufficient quality to predict RfDs for compounds lacking toxicological information. For compounds with known RfDs, 884 (79%) were within 8-fold of published RfDs, well within the acknowledged uncertainty inherent in published RfDs. Our approach has applicability beyond PIANO compounds and represents a new alternative methodology (NAM) that may be used to reduce uncertainty in human health risk assessment and guide regulatory decisions.

## 1. Introduction

Petroleum hydrocarbon products are mixtures often comprised of hundreds of aliphatic and aromatic compounds [1]. PIANO (Paraffin, Isoparaffin, Aromatic, Naphthene, and Olefin) analysis is an analytical tool widely used within the petroleum industry to characterize lightweight petroleum products (chemicals with boiling points of up to 250 °C) in drinking water. Specialized PIANO analyses can identify as many as 300 chemical constituents of JP-5 jet fuel in drinking water. Collectively, these chemical constituents may pose a risk of harm to human health when consumed in drinking water. While PIANO analyses can detect carcinogenic constituents (e.g., benzene), JP-5 does not contain significant concentrations of carcinogenic constituents [2]. As a result, JP-5 only has the potential to pose non-cancer health risks to people consuming JP-5 in drinking water.

The National Research Council (NRC) defined the paradigm currently used by the United States (U.S.) Environmental Protection Agency (EPA) [3,4] to characterize human health risk. Such an assessment integrates exposure and toxicity information to determine the aggregate of non-cancer risks posed by chemicals in the environment. An individual’s exposure is conservatively determined using EPA default exposure assumptions and EPA-derived chemical-specific toxicity values [5]. The EPA derives chemical-specific toxicity values following established EPA guidance for determining chronic oral reference doses (RfDs) and chronic inhalation reference concentrations (RfCs) [6]. The EPA’s process for deriving RfDs and RfCs is considered the scientific gold standard of chemical-specific toxicity value derivation. The EPA defines the RfD as “an estimate (with uncertainty spanning perhaps an order of magnitude) of a daily oral exposure to the human population (including sensitive subgroups) that is likely to be without an appreciable risk of deleterious effects during a lifetime” [6]. The RfC is similarly defined as “an estimate (with uncertainty spanning perhaps an order of magnitude) of the daily inhalation exposure to a chemical that is likely to be without an appreciable risk of deleterious effects during a lifetime of exposure” [6].

The EPA’s Integrated Risk Information System (IRIS) lists chemical-specific RfDs, in units of milligrams of compound per kilogram bodyweight per day (mg/kg-day) [7,8]. These RfDs are used in the assessment of human health risks posed by the consumption of contaminated drinking water. The IRIS also contains chemical-specific RfCs, reported in units of milligrams of chemical in a cubic meter (mg/m^3^) of air, for use in the assessment of human health risks posed by the inhalation of airborne contaminants. The IRIS lists 746 chemicals with either an RfD and/or an RfC. The Oak Ridge National Laboratories (ORNL) Risk Assessment Information Service (RAIS) also lists RfDs and RfCs [9]. The sources of these toxicity values include the EPA’s Provisional Peer-Reviewed Toxicity Values for Superfund (PPRTV) and those derived by various state agencies like the California Environmental Protection Agency (CalEPA) [9]. The RAIS lists 1142 unique chemicals with either an RfD or an RfC, including the 746 chemicals listed in the IRIS. Of these, 826 chemicals have an RfD and 316 chemicals have only an RfC.

The U.S. EPA Supplementary Guidance for Conducting Health Risk Assessment of Chemical Mixtures [10,11] describe two approaches for assessing human health risks posed by environmental mixtures: component methods and whole-mixture methods. Component-based approaches involve analyzing the toxicity of a mixture’s individual components and then selecting the most toxic compound’s toxicity value to represent that mixture or fraction. The EPA recognizes that this approach is fraught with uncertainty, and it is only recommended when appropriate toxicity data on a complex mixture, or on a sufficiently similar mixture, is not available. The U.S. EPA uses this approach to assess the non-cancer hazards of various aliphatic and aromatic Total Petroleum Hydrocarbon (TPH) fractions [12]. TPH fractions are generally classified into aliphatics and aromatics, and each of these major fractions are then further separated into low-, medium-, and high- carbon range fractions based on the number of carbon (C) atoms in the compounds and/or the compounds’ equivalent carbon (EC) number indexes. The EC index is related to the compounds’ potential transport in the environment and is equivalent to the retention time of the compounds in a boiling-point gas chromatography (GC) column (nonpolar capillary column), normalized to n-alkanes. Analytically, carbon range fractions are easily assessed as the total GC detection within the EC carbon range. For example, the C5–C8 aliphatic fraction is determined as the total amount of GC compounds detected between and including C5 and C8. Of those compounds within this fraction with published toxicity values, the EPA identifies cyclohexene as having the highest toxicity. The EPA identifies cyclohexene as the indicator compound for this fraction for use as the toxicological basis for a surrogate RfD applied to the C5–C8 aliphatic fraction.

The human health risk posed by exposure to petroleum mixtures is assessed as the sum of the mixture’s individual constituent health risks. Many of the constituent compounds in petroleum mixtures do not have either an RfD or an RfC. For these compounds, the EPA employs surrogate RfDs in risk assessment. The EPA assigned surrogate RfDs to groups of structurally similar compounds grouped into six Total Petroleum Hydrocarbon (TPH) fractions (Table 1). A surrogate RfD is often set equivalent to the most toxic compound in the TPH fraction [12]. For example, the compounds in the Aromatic Medium Carbon range (C9–C16) are assigned a surrogate RfD based on the toxicity of Trimethylbenzenes (95-63-6, 108-67-8, and 526-73-8), all of which share the same EPA RfD (Table 1). The use of surrogate RfDs in risk assessment adds uncertainty to risk estimates, since they tend to overestimate the human health risks posed by the compounds without RfDs. Identifying reasonably accurate RfD values for compounds without RfDs would greatly reduce the uncertainty inherent in human health risk assessments of JP-5 jet fuel PIANO target analytes in drinking water [10,12,13].

In silico computational tools, like our QSAR model, that forecast toxicity by correlating a chemical’s molecular structure and physicochemical properties with its oral toxicity endpoints act as timesaving, cost-effective alternatives to multi-generational mammalian exposure experiments traditionally used to determine mixture and mixture component toxicity. Our QSAR model can be used to predict the toxicity of petroleum mixture constituents lacking toxicological information and which are currently assessed for human health risk using surrogate toxicity values, which the EPA acknowledges are fraught with uncertainty. The use of predicted toxicity values for target petroleum constituents (e.g., target PIANO analytes) allows for complete and comprehensive use of the analytical data characterizing constituent compounds within the various petroleum fractions. This not only reduces the uncertainty associated with surrogate toxicity values use but also allows for the identification of individual petroleum hydrocarbon constituents as potential drivers of human health risk.

### Overview

Using a modification of the concepts and methods previously described [14], we predicted RfDs for PIANO compounds that do not already have EPA-published RfDs or RfCs. For compounds that lacked EPA-published RfDs but had published inhalation reference concentrations (RfCs), we calculated equivalent RfDs. Hereafter, both EPA-published RfDs and those calculated from RfCs are collectively referred to as “published RfDs”. Compound-specific 2-dimensional (2D) molecular descriptors (MDs) were obtained using the EPA’s Toxicity Estimation Software Tool (TEST) V 5.1.2 [15] for compounds with published RfDs and all PIANO compounds. We used a science and evidence-based computational approach to build five Quantitative Structure–Activity Relationship (QSAR) models to estimate RfDs for PIANO compounds. One of the prediction equations had statistical indicators of poor fit and was dropped. We predicted compound-specific RfDs as the geometric means of estimated RfDs generated from the remaining four QSAR models. Our predicted RfDs have the same acceptable precision (an order of magnitude) as those derived by the EPA and use more structural information (i.e., 797 MDs) than was applied by the EPA in the assignment of structurally similar surrogate RfDs. Using our predicted RfDs in human health risk assessment reduces the uncertainty inherent in the use of overly conservative EPA surrogate toxicity values and thereby results in a more accurate determination of human health risks. This process of determining RfDs using our method and QSAR models is consistent with the EPA’s “3R principles” to reduce, refine, and replace the use of animals in toxicological research [16].

## 2. Materials and Methods

### 2.1. Overview of the In Silico QSAR Model

Our approach for predicting chronic oral RfDs is based on the geometric means of the QSAR-model RfD estimates. Each of the QSAR models is based on stepwise multivariable linear regression analysis of MDs and published RfDs. Each QSAR model generated an RfD estimation equation, built using randomly generated training groups, consisting of 641 (80%) of the non-PIANO compounds and 21 (50%) of the 43 PIANO compounds with RfDs from the master dataset. To assess the equation quality, R^2^ and Q^2^ calculations were performed on the original training and testing sets and subcategories of PIANO compounds with published RfDs and non-PIANO compounds. For each QSAR model, t, the most optimal equation (discussed in Section 2.5) was identified and used to estimate RfDs for all compounds. The RfD estimate equation from one QSAR model, training set four, had low R^2^ and Q^2^ values and was dropped from the calculations. The remaining four QSAR model RfD estimates were used to calculate geometric means, resulting in our predicted RfDs.

The robustness of our approach was assessed by evaluation of precision and accuracy. The precision of the predicted RfDs is reflected by the narrow ranges of the distinct QSAR models’ RfD estimates. The accuracy of the QSAR models was demonstrated by comparing a predicted compound-specific RfD with its published RfD.

### 2.2. Oral RfD Master Dataset

A master dataset of RfDs was created using sources of published RfD values. Two of the largest and most respected of these databases are the Risk Assessment Information Service (RAIS) [9] and the Integrated Risk Information System (IRIS) [7]. To increase the number of compounds with RfDs from these datasets, an equivalent chronic oral RfD was derived for compounds with no RfD (mg/kg-day) but with an RfC (mg/m^3^). The derivation of an RfD from the RfC assumes route-specific dose equality. The RfD is determined by the product of the RfC and the default human breathing rate of 20 m^3^/day, divided by the default average human body weight of 70 kg. This is written as Equation (1):

RfD (mg/kg-day) = RfC (mg/m^3^) × 20 (mg/m^3^/day)/70 (kg). (1)

Extrapolating across exposure routes (i.e., ingestion and inhalation) has limitations based upon the physiochemical properties affecting absorption, distribution, metabolism and excretion, which are not accounted for in Equation (1) [17]. Regardless of this, the conversion of an RfC to an RfD, or more frequently the conversion of an RfD to an RfC, is routinely applied by regulators needing such values in risk assessment while fully understanding the attendant limitations. The chemicals for which we calculated RfDs from a published RfC are identified in Supplementary Table S1.

### 2.3. Molecular Descriptor Master Dataset

We identified 1390 compounds with unique Chemical Abstracts Service Registry Numbers^®^ (CASRNs) that had published RfDs or RfCs, or were among the PIANO compounds of interest. Each CASRN was entered into the EPA’s Toxicity Estimation Software Tool (TEST) to describe the compound’s 2D structure and provide 797 MDs based on that structure. The MDs, as specified in the Molecular Descriptors Guide [18], are based on the physical characteristics of the compound’s structure. Compounds were not included in the master dataset if the CASRN input into TEST resulted in an error. CASRN with TEST errors are identified in Supplementary Table S1. The resulting master dataset contained 1113 compounds: 290 PIANO compounds and 823 others. Of the original 290 PIANO compounds, 43 had published RfDs and 247 lacked RfDs. Nine PIANO compounds lacked MDs and were eliminated from further consideration in the QSAR model development. We identified 22 compounds with RfDs as being outliers (described below) and excluded them from further use in the QSAR model development (Supplementary Table S1). A total of 801 non-PIANO compounds with RfDs remained.

### 2.4. Identification of Statistical Outliers

**Figure 1:**
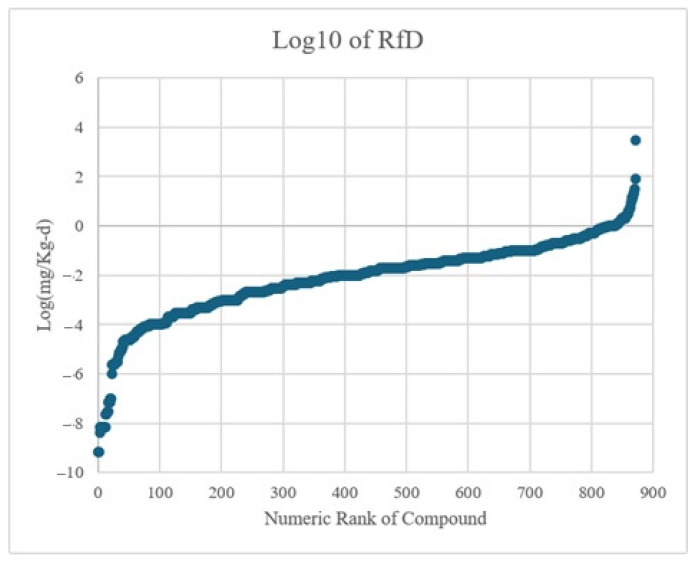
Distribution of logarithm base 10 of RfD values. RfD: chronic oral reference dose.

The distribution of published RfDs for the 866 compounds with MDs in the master dataset was clearly non-linear by visual inspection. The RfDs were transformed by log base ten, Log(RfD). When sorted by increasing value, the Log(RfD)s produced a sigmoid curve (Figure 1). The mean and standard deviation of these values were determined to be −2.133 and 1.588 respectively. Three standard deviations below the mean was −6.895 and above the mean was 2.630. This eliminated 21 compounds. The procedure was repeated with the 845 remaining compounds, yielding cut points of −5.931 and 1.952. This eliminated one more compound. The third iteration with cut points of −5.906 and 1.937 demonstrated no additional statistically extreme outliers. The twenty-two compounds in Table 2 were excluded from the QSAR model and RfD estimation equation generation.

### 2.5. Development of the In Silico QSAR Model

To reduce the number of MDs eligible for inclusion in the QSAR models, we used an approach parallel to one previously described [14]. Briefly, MDs with a higher proportion of zero values were excluded. The ones that were kept had non-zero values for at least 30% of each group of PIANO compounds, non-PIANO compounds excluding outliers, and non-PIANO compounds with outliers. This left 316 (40%) of the original 797 MDs for consideration.

To mitigate the risk of chance correlations, five training sets were generated by random selection of 640 (80%) of the 801 non-PIANO compounds (excluding outliers) and 21 (50%) of the 43 PIANO compounds containing RfDs. Stepwise multivariable linear regression analysis was performed on each training group. Different combinations of values between 0.05 and 0.15 were used for significance levels to enter (SLEs) the model and significance levels to stay (SLSs) in the model for each training group. The estimation equations in this method, like those previously developed [14], rely only on correlations between MDs and published RfDs. No other characteristics of chemical-specific RfDs and RfCs were used in the QSAR model development. All analyses were conducted using SAS version 9.4, Cary, NC 27513 and Excel for Microsoft 365, Redmond, WA 98052.

### 2.6. Identification of Most Optimal In Silico QSAR Models

**Figure 2:**
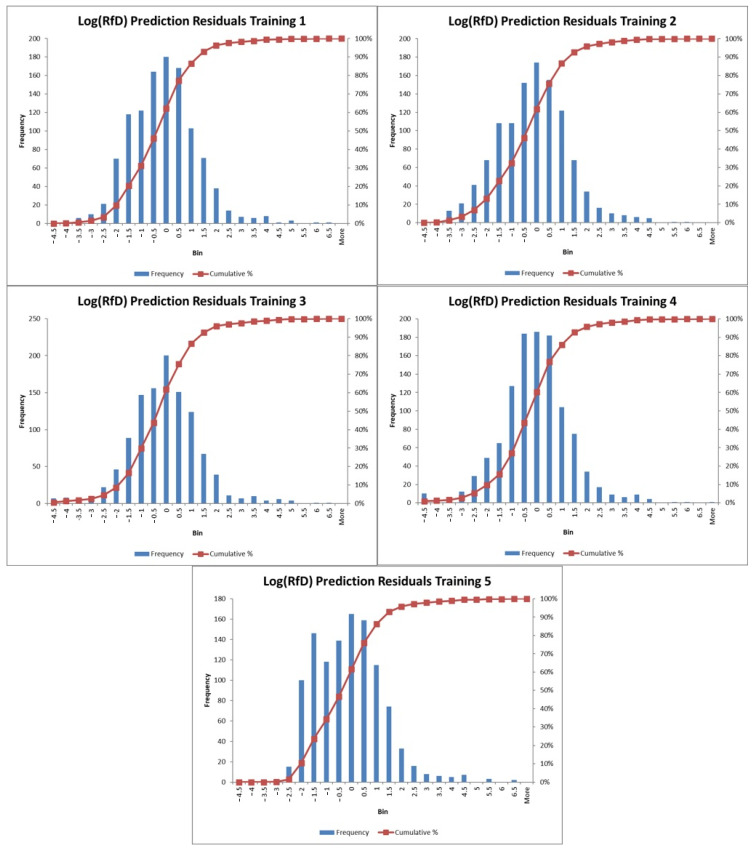
Prediction residuals. The frequency of occurrences by range is displayed on the left axis. The cumulative percentage of occurrences is displayed on the right axis.

**Figure 3:**
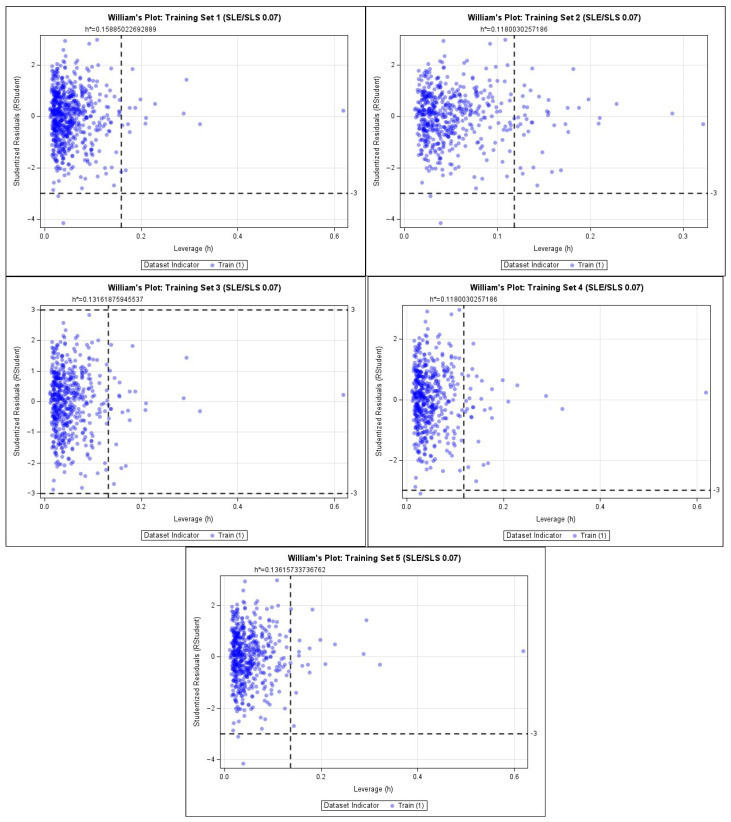
William’s plots for training sets 1–5. SLE: the significance level to enter, meaning a variable must have a p-value smaller than this to be added to the regression equation; SLS: the significance level to stay, meaning a variable must have a p-value smaller than this to be retained in the regression equation; h*: the warning leverage, representing the statistical cutoff point desired to avoid excessive influence from a given compound; Train (1): the variable that indicates if a compound is in the training set (1) or not (0).

For each training set, a family of five models was created with differing SLE and SLS significance level combinations (i.e., 05 05, 07 07, 09 09, 11 11, and 15 15, respectively). The most optimal model from the family of equations was selected by considering multiple counterbalancing factors. The selection was intended to identify the most suitable number of appropriate MDs, with less underfitting with too few MDs and overfitting with too many MDs, as discussed in statistics references [19,20]. Multiple factors were considered in the selection of optimal equations. These included root mean square errors (RMSEs), R^2^ and Q^2^ results, and their ratios. These were calculated for the groups of all compounds, only PIANO compounds, and only non-PIANO compounds. We also reviewed residual (Figure 2), leverage, Mallow’s Cp statistic, and graphic results to include William’s plots (Figure 3). In cases of equations with similar predictive power, we selected the more parsimonious equations with fewer MDs. The most optimal equations were seen when SLEs and SLSs were 0.07, containing 25 to 34 MDs. The MDs and betas from the 25 regressions are available in Supplementary Table S2. Equation (2) is a sample equation produced by the model. MDs and their definitions from the five models are provided in Supplementary Table S3. More details are available from the EPA’s Molecular Descriptor Guide [18].

Log(Rfd) = −8.678997284 − 1.690078988(ATS1m) + 0.855054927(BELe1) + 6.664968657(CID2) + 0.128323455(Gmax) − 0.111211976(Hy) − 0.099029647(Jt) + 0.095971061(Qs) − 0.210987579(XLOGP) + 0.123744065(ka1) − 3.077952211(piPC01) + 0.191343947(BEHm2) − 0.585353052(BEHe3) + 1.688862744(ATS2e) + 0.396997763(MATS2p) + 0.28761101(BEHm4) − 0.636152818(piPC03) + 0.383480705(MATS3e) + 0.228309046(piPC05) + 0.270307396(MATS5m) − 0.211380267(MATS6v) + 0.145864045(nBM) − 0.386895525(nBnz) + 0.737578774(BELp8) − 0.954586492(xp10) − 0.353586402(MATS8e)(2)

Additionally, we performed regression analyses on subsets of similar compounds in the master list to assess the quality of our models’ results. These subsets excluded halogenated and nitrogen- and sulfur-substituted compounds. The initial evaluation involved all compounds with RfDs. The second evaluation excluded any compounds containing halogens. The third evaluation additionally excluded compounds containing nitrogen or sulfur. We found that the most accurate and precise predictions of compound-specific RfDs occurred when more compounds were included in the model development regardless of the specific elements they contained. Results are only reported for this full set. Determination of the consistency of the results with stepwise inclusion showed that 238 (75%) of the 316 MDs were not in any of the optimal models. Of the 78 MDs identified in the 5 optimal QSAR models, consistency was demonstrated by the identification of 3 MDs (4%) (ATS1m, Jt, and MATS5m) in all five models, 13 MDs (17%) in at least 4 models, and 18 MDs (23%) in at least 3 models.

## 3. Results

### 3.1. Model Validation and Applicability Domain

The root mean square errors were slightly less than 1. Since this value is linked to Log(RfD), it would suggest that, on average, the predicted RfDs would be no more than an order of magnitude different. The R^2^ values from the most optimal identified QSAR models from each of the five training sets ranged from 0.4416 to 0.5009. Adjusted R^2^ values ranged from 0.4196 to 0.4738. We considered these to reflect moderate fit. As mathematically expected, Q^2^ values were lower. Model 4, with a Q^2^ of 0.0593 and a Q^2^-to-R^2^ ratio of 0.1254, indicated poor predictive ability and was subsequently removed from use in the determination of predicted RfD values. The remaining models had Q^2^ values of acceptable model quality, between 0.1779 and 0.4128, which we interpret as suitable.

The low Q^2^ for Model 4 was concerning. Its estimates derived from the fourth QSAR model equation were consistently high for the low and medium aliphatic and low and medium aromatic compounds. The exclusion of model 4 resulted in a dramatic narrowing in the range of RfD estimates. The selected QSAR model equations produced RfD estimations that were most similar to published RfDs, regardless of whether the compound was in the training group or testing group. The statistics for the best equation from each training set are displayed in Table 3. The last two rows of Table 3 reflect fit when stratified by PIANO and non-PIANO compounds. Further stratification resulted in groups that were too small to produce meaningful, reliable statistics.

The QSAR model equation residual errors (the difference between published and estimated Log(RfD) values) were analyzed to check for systematic errors. Histograms representing the residual errors and plots of residuals versus predicted values are provided in Figure 2. These plots show that, in general, the residual errors are normally distributed and randomly scattered around zero. Each QSAR model equation training set provided reasonably good predictive power, generating estimated RfDs for compounds in the master dataset.

### 3.2. Precision and Accuracy Assessment

We identified an optimal equation for each of the five QSAR models from training sets randomly selected from the master dataset. The fourth QSAR model equation with poor fit was eliminated from further use in predicting compound-specific RfDs. We predicted compound-specific RfDs as the geometric means of the remaining QSAR model equation RfD estimates.

The precision of predicted compound-specific RfDs is depicted by the range of four QSAR model equation compound-specific RfD estimates. Where a compound had an accepted RfD, accuracy was assessed as the ratio of the predicted to the published RfD. Ratios exceeding an order of magnitude suggested poor predictive ability. However, such ratios can be strongly influenced by the information used to determine the published RfD. Specifically, the derivation of published RfDs often includes the use of uncertainty factors (UFs) to lower the resulting RfD by as much as 3000 times below the applicable human equivalent dose associated with the critical effect. UFs are used to adjust the RfD downward by a factor of from 3 to 10 each to include the most sensitive human population and to account for uncertainty in extrapolating from the dose causing the critical effect in animals to one in humans, to extrapolate from a subchronic to a chronic dosing study, to extrapolate from a Low Observed Adverse Effect Level (LOAEL) to a No Observed Adverse Effect Level (NOAEL), and to reflect on the completeness of the dataset available for RfD derivation (e.g., whether the dataset is missing neurotoxicology, immunotoxicology, reproductive, and/or developmental studies). Our QSAR model equations only use MDs and published RfDs, and do not consider the details of RfD derivation (e.g., the critical effect and UFs used). Because we used all published RfDs in our model development, which covered all potential critical effects and UFs, our predicted RfDs are not constrained by the details of RfD derivation. Consequently, where published RfDs employed large composite UFs, the ratio of the predicted to the published RfD may exceed an order of magnitude simply because of the uncertainty inherent in the derivation of the published RfD. The uncertainty inherent in our QSAR model equations is limited to within an order of magnitude for most compounds. The difference between our predicted RfDs and the published RfDs may in part stem from the use of UFs in the derivation of published RfDs. For example, where a compound has not been assessed for reproductive toxicity, the RfD is based on some other critical effect (e.g., liver toxicity) and a UF of 10 is applied to the RfD to account for the uncertainty associated with not having performed that reproductive toxicity assessment. Our predicted RfD is not hampered by such uncertainty since it is based on all published RfDs, including those derived from critical reproductive effects. Consequently, where our model predicts an RfD that is more than an order of magnitude lower or higher than the published RfD, we might critically review the uncertainty factors applied to its derivation.

Where our compound-specific RfD prediction differs by more than an order of magnitude from the published RfDs, we recommend further toxicological study. Our process for developing QSAR model equations offers an opportunity for additional research into the selection of critical effects and the use of UFs. Clearly, the identification of MDs associated with certain critical effects can be used to direct toxicological assessment of compounds. Our process for using published compound-specific RfDs and MDs to predict chronic oral RfDs lends itself to further research into the process of deriving these RfDs, including the selection of critical effects, dose metrics, effect severity, and how uncertainty is addressed.

### 3.3. Overall Assessment

All QSAR models demonstrated comparable RfD estimates for PIANO compounds. The range between the lowest and highest of the four QSAR model RfD estimates, an indicator of precision, generally remained narrow, indicating high precision. For the 290 PIANO compounds, 240 (83%) had ranges < 10-fold. Out of all 1113 compound (including PIANO and outliers) predictions, 948 (85%) had ranges < 10-fold. This includes all compounds with MDs. As described later, the high-carbon (C30–C40) linear aliphatics were the only notable exception, with a wide range of RfD estimates. This is likely a consequence of having no RfDs for high-carbon linear aliphatic compounds for the QSAR models to use with MDs in equation building, with the highest carbon count occurring with n-Nonane (111-84-2) (C9).

As expected, as the number of compounds in a training group increased, optimal equations required more MDs. At the same time, to minimize the number of MDs in the QSAR models, the significance level required for the MDs to remain in a QSAR model equation needed to decrease (be more exclusive).

Although the MDs and MD coefficients identified in the QSAR model equations varied among the four different training groups, estimated RfDs remained comparable. It is important to note that larger MD coefficients do not necessarily imply a greater influence on RfD estimates than those MDs with smaller coefficients. Compound-specific toxicity within any particular organism or cell system can be approximated by its biochemical properties, such as a specific functional group or electronegativity. Where an MD more accurately represents the causal mechanism of toxic action, that MD can have a small coefficient and a significantly higher impact on a model’s predicted toxicity value than an MD, not casually related to the toxic mechanism of action, that has a large coefficient. The full list of predicted and published RfDs for the PIANO compounds is provided in Supplementary Table S4. The MDs used in our analysis are available in Supplementary Table S5.

### 3.4. Comparisons of Predicted and Surrogate RfD Values

**Figure 4:**
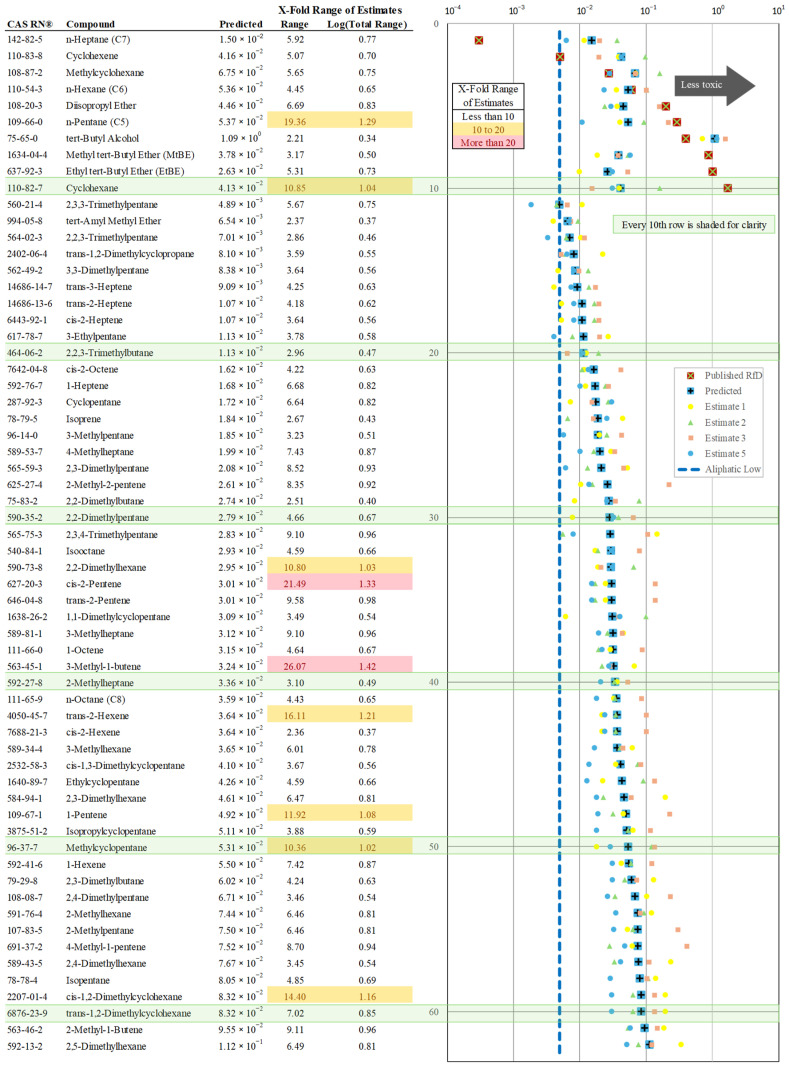
Aliphatic Low Carbon (C5–C8) fraction (mg/kg-day). Comparison of results from four QSAR equations (estimates 1–4) and their geometric means (predicted blue squares) with U.S. EPA Surrogate RfD (Cyclohexene, 5.00 × 10^−3^) (blue dashed line) and published RfDs (red squares). QSAR Model 4 estimates were dropped due to low R^2^ and Q^2^ values. CAS RN^®^: Chemical Abstracts Service Registry Number, an unambiguous, unique compound identifier.

We compared predicted RfDs with EPA surrogate RfDs for Aliphatic Low (C5–C8), Aliphatic Medium (C9–C18), Aliphatic High (C19+), Aromatic Low (C6–C8), Aromatic Medium (C9–C16), and Aromatic High (C17–C32) (Table 1). Figure 1, Figure 2, Figure 3 and Figure 4 are grouped by surrogate category and display published; estimated; predicted; and, where applicable, surrogate RfDs for each PIANO compound.

### 3.5. Aliphatic Low Carbon (C5–C8) Fraction

In Figure 4, the surrogate value of 5.0 × 10^-−3^ mg/kg-day (blue dashed line) is based upon the published RfD for the olefin Cyclohexene (110-83-8). The range of predicted RfDs for the 62 compounds of the Aliphatic Low Carbon fraction (4.89 × 10^−3^ to 1.09 × 10^0^ mg/kg-day) was comparable to that of the published RfDs (3.01 × 10^−4^ to 1.71 × 10^0^ mg/kg-day). Every predicted RfD was at or above the surrogate value (i.e., less toxic). Seven out of 62 RfD predictions (11%) were within a factor of ten of their published RfDs. The predicted RfD for Cyclohexane (110-82-7), a naphthene or cyclic, fully saturated aliphatic, was lower (more toxic) than the published RfD by a factor of 11. The average of the ranges of estimates was 6.7. The ranges exceeded 20-fold in 2 out of the 62 compounds (3%) (highlighted in pink). The maximum range of estimates was 26-fold, less than two orders of magnitude. Three RfD predictions were moderately lower (more toxic) than their published RfDs: Cyclohexane (110-82-7), Ethyl tert-Butyl Ether (ETBE) (637-92-3), and Methyl tert-Butyl Ether (MTBE) (1634-04-4). These compounds had respective predicted RfDs that were 41-, 38-, and 23-fold lower (more toxic) than their published RfDs.

### 3.6. Aliphatic Medium/High Carbon (C9+) Fractions

**Figure 5:**
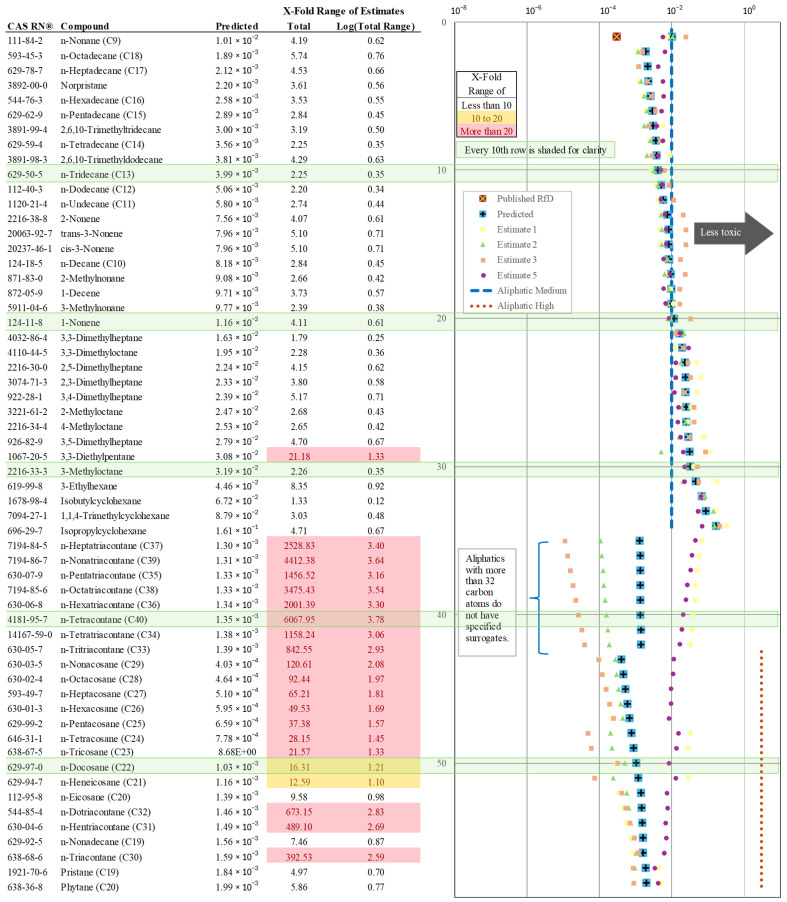
Aliphatic Medium/High Carbon (C9+) fractions (mg/kg-day). Comparison of results from four QSAR equations (estimates 1–4) and their geometric means (predicted blue squares) with U.S. EPA surrogate toxicity values. These values for the Aliphatic Medium Carbon (C9–C18) fraction are based upon mid-range aliphatic streams (<1% aromatics) (1.00 × 10^−2^) (blue dashed line), and for the Aliphatic High Carbon (C19–C32) fraction they are based on white mineral oil (3.00 × 10^0^) (brown dotted line). Estimate 4 has been dropped due to low R^2^ and Q^2^ values. The CAS RN (Chemical Abstracts Service Registry Number) is an unambiguous unique compound identifier.

In Figure 5, the surrogate chronic oral RfD for the Aliphatic Medium Carbon fraction is 1.0 × 10^−2^ mg/kg-day based on the published RfD toxicity value associated with mid-range aliphatic streams. n-Nonane (111-84-2) is the only compound in this fraction with a published RfD. Briefly, the chronic oral RfD for n-nonane published by RAIS is 3.0 × 10^−4^ mg/kg-day. It is described by the RAIS as the subchronic RfD determined by the U.S. EPA as a Provisional Peer-Reviewed Toxicity Value (PPRTV) of 3.0 × 10^−3^ mg/kg-day divided by an additional uncertainty factor of 10 to reflect chronic dosing [21]. The U.S. EPA declined to identify a chronic oral RfD because of the high level of uncertainty in the derivation of a subchronic oral RfD (i.e., the composite UF was 3000). The U.S. EPA did identify a chronic inhalation reference concentration (RfC) of 2.0 × 10^−2^ mg/m^3^, which can be converted, as described earlier, to a chronic oral RfD of 5.7 × 10^−3^ mg/kg-day. Both chronic oral RfDs for n-nonane are lower than the surrogate RfD of 1.0 × 10^−2^ assigned to this petroleum fraction, but the published RfD for n-Nonane has an uncertainty spanning 30,000-fold.

The RfD predictions for the compounds within this carbon fraction range from 1.89 × 10^−3^ to 1.61 × 10^−1^ mg/kg-day and include 18 out of the 34 RfD predictions (53%), being slightly lower (more toxic) than the surrogate RfD by no more than a factor of 5.3. The range (21) on one estimate was notably larger than the others. The average of the other 33 was 3.6-fold.

The surrogate RfD for the Aliphatic High Carbon fraction, including the linear aliphatics up to C32 is 3.0 × 10^0^ mg/kg-day (brown dotted line). This is based on the published RfD for white mineral oil. All the predicted RfDs in the Aliphatic High Carbon fraction are substantially (up to six orders of magnitude) lower (more toxic) than the EPA-published RfD for white mineral oil, the surrogate RfD for this carbon fraction. The ranges of estimated RfDs for the longer linear aliphatics (>C29) span multiple orders of magnitude, suggesting the need to interpret our RfD predictions with caution in this domain.

The substantial difference between the published RfD for white mineral oil and the predicted RfDs for white mineral oil and the other members of the Aliphatic High Carbon fraction may be partially explained by the fact that the compounds in this petroleum fraction have very low gastrointestinal (GI) bioavailability. In human health risk assessments, the EPA generally assumes that the default GI absorption of constituents of the Aliphatic High Carbon fraction is 100%. Bioavailability is not applied to RfD derivation but should be considered when determining the Point of Departure (POD) during RfD derivation and then when applying an RfD to the assessment of human health risk posed by oral ingestion. White mineral oil and the other constituents in the Aliphatic High Carbon fraction have very low oral bioavailability [21,22]. The RfD for white mineral oil is based on its therapeutic use in human children as a laxative. This therapeutic dose was not adjusted to reflect the fact that less than 2% of the administered oral dose was absorbed into the body [23,24]. The published RfD for white mineral oil does not reflect the absorbed dose, but the total therapeutic dose, much of which was eliminated with feces and never absorbed into the body. Consequently, the published RfD for white mineral oil is nearly 100 times greater (less toxic) than if the absorbed dose had been correctly used as the defining POD in RfD derivation.

Our QSAR model equations do not consider the potential human GI bioavailability of compounds from drinking water. Furthermore, we do not know if white mineral oil may have a higher toxicity if injected into humans. We speculate that the much higher surrogate RfD (lower toxicity) reflects the very low bioavailability of white mineral oil and that this might reasonably be expected of the constituents of the Aliphatic High Carbon fraction. This example addresses the model applicability domain. Our models should not be used where this level of imprecision exists.

### 3.7. Aromatic Low (C6–C8) and Medium (C9–C16) Carbon Fractions

**Figure 6:**
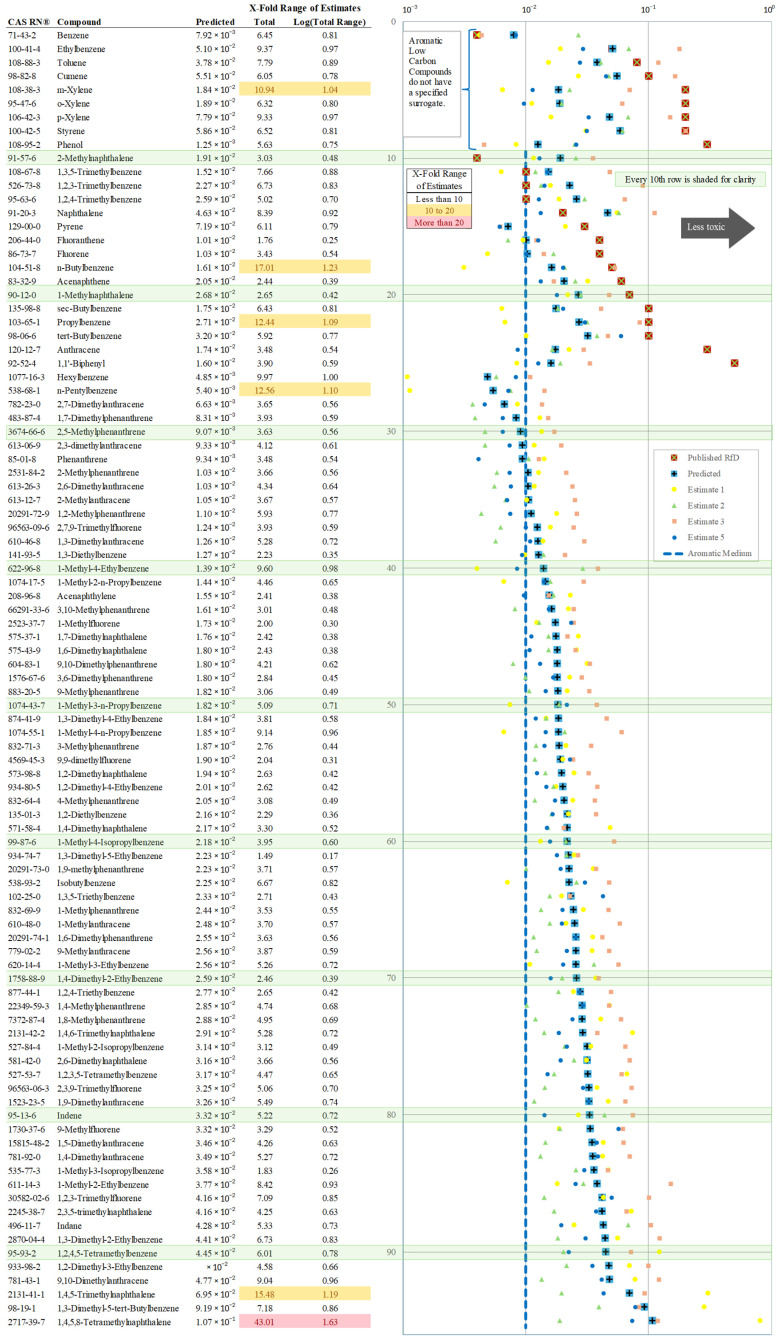
Aromatic Low (C6–C8) and Medium (C9–C16) Carbon fractions (mg/kg-day). Comparison of results from four QSAR equations (estimates 1–4) and their geometric means (predicted blue squares) with U.S. EPA-published RfDs (red squares). For the Aromatic Medium Carbon fraction (C9–C16), the U.S. EPA surrogate toxicity value is based upon Trimethylbenzenes (1.00 × 10^−2^) (blue dashed line). Estimate 4 has been dropped due to low R^2^ and Q^2^ values. CAS RN^®^: Chemical Abstracts Service Registry Number, an unambiguous, unique compound identifier.

In Figure 6, most predicted RfDs align well with the published RfDs in the Aromatic Low and Medium Carbon fractions, with only five (16%) differing by more than a single order of magnitude. Phenol (108-95-2) was surprising with a predicted RfD of 1.25 × 10^−2^, which was 24-fold lower (more toxic) than the published RfD of 3.00 × 10^−1^. The greatest difference between predicted and published RfDs in the Aromatic Medium Carbon fraction was seen with 1,1′-Biphenyl (92-52-4), which was 31-fold lower (more toxic) than its published RfD. The surrogate RfD for the Aromatic Medium Carbon group of 1.0 × 10^−2^ mg/kg-day, based upon Trimethylbenzenes (95-63-6, 108-67-8, and 526-73-8), is the same or lower (more toxic) than 73 out of the 86 compounds (85%). The most toxic predicted RfD for Trimethylbenzenes belongs to 1,3,5-Trimethylbenzene (108-67-8), which is only 1.5-fold higher (less toxic) than the surrogate RfD. This is well within the acceptable range that published RfDs may vary. The highest and lowest of the three predictions for Trimethylbenzenes differ by a factor of 1.7. The range of RfD predictions for the Aromatic Low and Medium Carbon fractions (4.85 × 10^−3^ to 1.07 × 10^−1^ mg/kg-day) closely matches the range of published RfDs (4.0 × 10^−3^ to 5.0 × 10^−1^ mg/kg-day). Out of the 25 compounds with published RfDs, the maximum fold increase was 5 for 2-Methylnaphthalene (91-57-6). The ranges in estimated RfDs are less than 6-fold for 67 (71%) of these compounds.

### 3.8. Aromatic High Carbon (C17–C32) Fraction and Others

**Figure 7:**
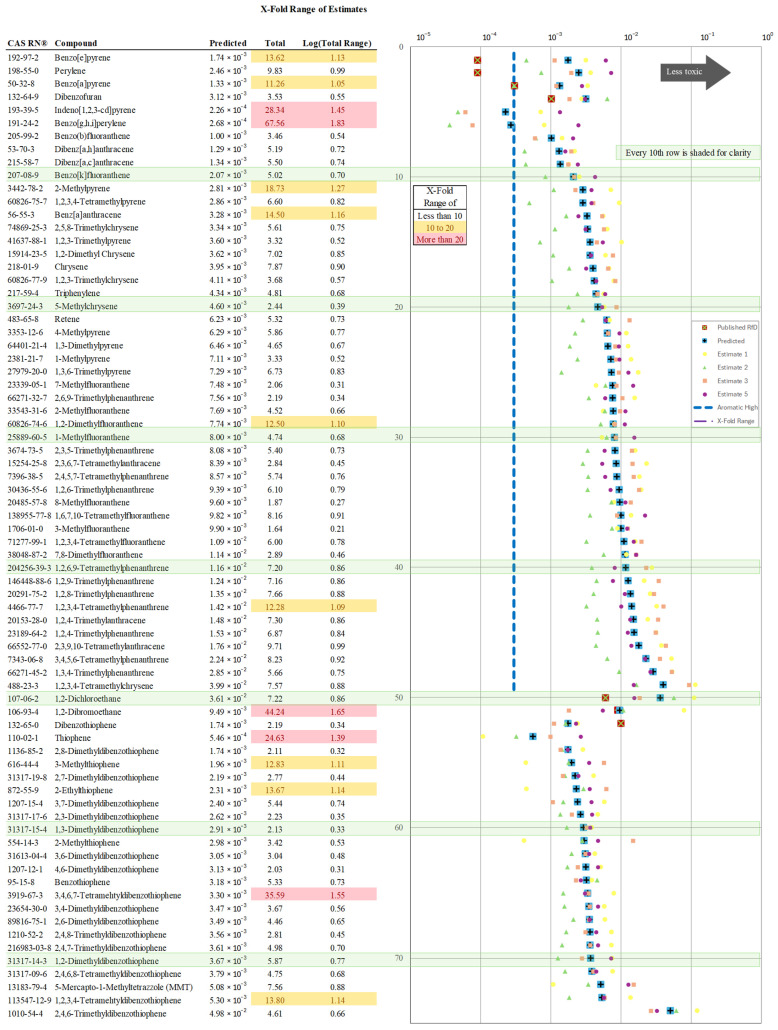
Aromatic High Carbon (C17–C32) fraction and others (mg/kg-day). Comparison of results from four QSAR equations (estimates 1–4) and their geometric means (predicted blue squares) with the U.S. EPA surrogate toxicity value of Benzo[a]pyrene (3.00 × 10^−4^) (blue dashed line) and published RfDs (red squares). Estimate 4 has been dropped due to low R^2^ and Q^2^ values. CAS RN^®^: Chemical Abstracts Service Registry Number, an unambiguous, unique compound identifier.

In Figure 7, the surrogate RfD for the Aromatic High Carbon fraction is 3.0 × 10^−4^ mg/kg-day. This surrogate RfD is based upon the published RfD for Benzo [a]pyrene (50-32-8). All but 2 out of the 49 predicted RfDs (4%) are higher (less toxic) than this surrogate RfD. One of these is the predicted RfD of 2.68 × 10^−4^ for Benzo [g,h,i]perylene (191-24-2). The other predicted RfD is 2.26 × 10^−4^ mg/kg-day for Indeno [1,2,3-cd]pyrene (193-39-5). Both are essentially the same as the surrogate RfD. Both the Benzo [e]pyrene (192-97-2) and Perylene (198-55-0) published RfDs were lower (more toxic) than the surrogate RfD, while our predictions were higher (less toxic) than the surrogate RfD. Aromatic High Carbon compounds’ RfD estimates exceeded 20-fold in 3 out of the 49 (6%) compounds. No compounds had RfD estimate ranges that reached two orders of magnitude.

Published RfDs were available for only 3 out of the 25 “other” compounds. All were within a factor of six from their published RfDs. In this group of 25 compounds, Thiophene (110-02-1) is the only compound with a predicted RfD (5.46 × 10^−4^ mg/kg-day) lower than any other published RfD within the group. Dibenzofuran (132-64-9) has a published RfD of 1.0 × 10^−3^ mg/kg-day, which is higher by a factor of three (less toxic).

## 4. Discussion

### 4.1. Quality of Model

#### 4.1.1. Predicted Precision and Accuracy Versus Published RfDs

Ratios of predicted to published RfDs near unity indicate a similar magnitude. Ratios larger than unity indicate a predicted RfD greater than the known RfD, suggesting lower toxicity than the published RfD. In contrast, ratios smaller than unity indicate a predicted RfD smaller than the published RfD, suggesting higher toxicity than the published RfD. The precision demonstrated by this approach (as measured by the narrow range of most QSAR model-estimated RfDs) and the accuracy (as demonstrated by the close approximations of predicted RfDs with published RfDs) provide confidence that predicted RfDs can be used as interim, tentative, or working non-cancer toxicity values in the assessment of human health risk. Since published RfDs have, by definition, uncertainty spanning perhaps an order of magnitude, it seems reasonable to accept predicted RfD values when the range of the QSAR model RfD estimates does not exceed an order of magnitude.

#### 4.1.2. QSAR Model Validation and Justification

Our QSAR models comply with each of the first four out of the five OECD identified validation principles [25,26]. Our QSAR clearly predicts an environmental effect, the compound-specific RfD derived from compound-specific physical, chemical, and biological effects with an estimate of uncertainty acceptable for RfDs, an order of magnitude. The RfD is a toxicological endpoint defining the condition of exposure above which we have heightened concerns that exposed people will develop adverse health outcomes. Our QSAR uses a transparently documented mathematical/statistical method to relate chemical structures (2-dimensional molecular determinates) to the biological activity/property, the published RfD. Our QSAR model also defines an applicability domain that includes all published RfDs in the IRIS and RAIS databases and all the 797 compound-specific 2D MDs that can be statistically linked to RfD estimates. The applicable domain is limited to compounds that strictly follow the prescribed RfD derivation process. We provide an internal validation and a statistical determination of our QSAR robustness using the training set and an external validation of how accurately our model predicts RfDs within a validation set, which is discussed later in this section in more detail. Finally, we are unable to provide a physical, chemical, or biological link between the MDs used and the predicted RfDs. However, a lack of mechanistic insight does not automatically disqualify our model. While documentation of plausible mechanistic associations significantly increases confidence in the model’s predictions, it cannot be used to invalidate our ability to predict RfDs for compounds lacking toxicological information. In fact, the results of our QSAR model can be used to prioritize and focus toxicological research.

Our process for using the geometric mean of four QSAR model estimated RfD values provides reasonably accurate RfD predictions that do not differ by more than an order of magnitude from published RfDs. Our model is based on a QSAR relationship derived from only published RfDs and MDs and was developed without consideration of any other parameters used in the derivation of RfDs (e.g., the selection of the critical effect and the use of UFs) [7]. Specifically, we use all published RfDs in the QSAR model development. This covers all potential critical effects used to derive chronic oral RfDs. As a result, our predicted RfDs are not constrained by the selection of the critical effect associated with published RfD derivation. The large number of published RfDs provides an ample base from which to predict an RfD based just upon MDs. Implicit in our predicted RfD is the assumption that the critical effect is well represented within the published RfD values. Our predicted RfD is not hampered by such uncertainty since it is based on all published RfDs, including those derived from critical reproductive effects. Consequently, our model’s RfD prediction includes potential reproductive toxicity described by certain MDs. Where our predicted RfD is more than an order of magnitude lower or higher than the published RfD, we recommend critical review of the critical effect selected and the use of uncertainty factors applied to its derivation.

Where published RfDs have employed large composite UFs, the ratio of the predicted to the published RfD may exceed an order of magnitude simply because of the uncertainty inherent in the derivation of the published RfD (e.g., n-Nonane). The derivation of published RfDs often includes the use of several UFs to account for uncertainty in the derivation of a safe dose. These UFs are associated with the extrapolation from the dose causing the critical effect in animals to one in humans, extrapolation from a subchronic to a chronic dose, extrapolation from a LOAEL to a NOAEL and accounts for the completeness of the dataset available for RfD derivation.

Our QSAR model equations do not consider these potentially relevant details of RfD derivation (e.g., the selection of the critical effect and UFs used), and yet the uncertainty inherent in our predicted RfD for most compounds is limited to within an order of magnitude. This likely stems from the uncertainty (i.e., use of UFs) in the derivation of published RfDs, which can be as much as 3000-fold or more. Our process for developing QSAR model equations offers an opportunity for research into the reliance of RfDs on the selection of critical effects and how such toxicity values vary with the use of UFs. We think that if we identify certain MDs with certain critical effects, the information derived could be used to focus toxicological assessment of compounds based upon their MD representation. We strongly recommend further toxicological study of any compound-specific RfD prediction that differs by more than an order of magnitude from its published RfD.

The ability of our approach to predict RfDs for most compounds with an uncertainty of less than an order of magnitude gives us confidence that the predicted compound-specific RfDs can be used in the assessment of human health risk. The use of these predicted RfDs instead of the surrogate RfD values currently employed promises to significantly lower the uncertainty inherent in the use of these surrogate toxicity values. The precision and accuracy of our approach to RfD derivation for compounds with published RfDs is a testament to the consistent use of the U.S. EPA’s RfD derivation process.

We used 2D MDs in our QSAR model because they are particularly useful in screening large, structurally diverse databases to predict general toxicity endpoints like the RfD. The lack of available specific receptor-binding data precludes the use of 3D MDs, which would be more useful for modeling mechanism-driven toxicity pathways. Our decision to use 2D MDs is more consistent with the expectation that RfD values may not always arise from known receptor-mediated mechanisms of action (MOAs). While 3D MDs, which typically have a higher information content with richer spatial and quantum-chemical data, might provide higher QSAR model resolution (reduce prediction error), 2D MDs are a robust option for capturing topological and physicochemical properties sufficient for use in predicting health effects (e.g., RfDs) from diverse datasets with high interpretability (i.e., specific alerts for structural toxicology). The use of 2D MDs is also computationally efficient and does not require the alignment typically required in 3D model assumptions regarding specific 3D bioactive conformations. Finally, the use of 2D MDs in QSAR models is generally associated with less noise and avoids the “overfitting” that can arise from highly complex 3D MDs that may not correspond directly to toxicity endpoints. 2D MDs are unable to distinguish between stereoisomers (e.g., cis/trans and R/S configurations), which can have very different toxicities, and fail to account for spatial nuances (like steric hindrance) that are often crucial for modeling receptor binding. In toxicology, however, we do not always know or appreciate the MOA governing the appearance of the critical toxicological effect used in RfD derivation. With these considerations, the use of 2D MDs is an acceptable limitation on our QSAR model output.

Future QSAR models might use 3D MDs to capture spatial and geometric properties, such as molecular volume, surface area, and steric/electrostatic fields, which can be useful in receptor interaction mapping, and can inform possible MOAs based upon chemical–receptor interactions. The use of 3D MDs can also improve the QSAR model’s ability to distinguish between different isomers (e.g., xylene molecules), capturing the nuanced shape-dependent differences in toxicity.

### 4.2. Application of QSAR-Predicted RfDs

#### 4.2.1. Comparison with Surrogate Values

The accuracy and precision of our predicted RfDs support serious consideration of their use in the characterization of the human health risk posed by these compounds. Using the most toxic compound’s RfD as a surrogate toxicity value for structurally similar compounds lacking toxicity information likely leads to an overestimation of human health risk. Having a more accurate determination of compound toxicity would result in more accurate human health risk estimates. On the other hand, using our approach to predict RfDs for compounds without published RfDs could potentially lead to underreporting of compound toxicity, as illustrated by *n*-Nonane (111-84-2). But it is also true that our predicted RfDs are likely more accurate than some published RfDs. Compounds like n-Nonane can include significant uncertainty in their RfD derivation (i.e., 30,000-fold). The use of our process for predicting RfDs can also be used to assess the impact of critical effect selection and to inform risk managers of the potential uncertainty in published and predicted RfDs. Clearly, our process for predicting RfDs can be used to identify compounds for additional focused toxicological assessment. Such assessments promise to further reduce the uncertainty inherent in the toxicity values used in human health risk assessment.

Intuitively, using a larger training dataset in QSAR model development or employing many more QSAR model outputs would likely result in improved model precision and accuracy in predicting RfDs. Where our approach predicted smaller RfDs (more toxic) than the surrogate RfDs, we strongly recommend prioritizing focused toxicological assessment of these compounds for the purpose of defining the uncertainty inherent in the RfD derivation process, e.g., *o*-xylene (95-47-6).

Our approach for predicting compound-specific RfDs, as the geometric mean of four optimized QSAR equations, minimizes the potential impact of any one model and its training set. Larger training sets and the use of more models would clearly reduce the uncertainty inherent in the predicted RfDs. Optimization is important to limit problems with underfitting and overfitting. Sometimes the optimal equation for a given training set shows that its performance in prediction outside of the training set is poor such that it should be discarded. When evaluated statistically, it was noted that optimal equations typically had a Mallow’s C_p_ value of about twice the number of MDs selected for use in the QSAR model equation. An example showing this is available in Supplementary Table S3.

For a variety of reasons, our QSAR model is not capable of predicting a useful RfD for all target PIANO analytes. Reasons to have low confidence in our QSAR model output include limited representation of published RfDs for specific petroleum fractions (e.g., the Aliphatic High Carbon faction), derivation of published RfDs using “considerations” not specified in applicable procedural guidance (e.g., outliers), and errors in RfD derivation (e.g., white mineral oil). For the majority of the nearly 300 target PIANO analytes (79%), our model provides reasonably accurate predictions of compound non-cancer toxicity (i.e., RfDs). This suggests that a similar approach could be used to predict RfDs for other compounds with little or no toxicological information (e.g., organic compounds used as food additives, pharmaceuticals, and pesticides).

Our analysis of residuals and statistical fit indicates that our QSAR model performs reasonably well in predicting RfDs for compounds lacking toxicity information. However, we cannot know exactly how well our QSAR model performs (i.e., its precision and consistency) without being able to compare its output with some quantitative toxicity value (e.g., a published RfD) in which we have high confidence. We are not aware of additional published and verified toxicity information (e.g., RfDs) that we could use to further refine our QSAR model results in this domain. While future QSAR model refinement could include inclusion of the critical toxicological effects and uncertainty factors (UFs) applied in RfD derivation, the increased granularity provided by these parameters would likely reduce the power of the resulting QSAR model to predict either the critical toxicological effect, the inherent uncertainty, or the RfD for compounds lacking toxicological information. Furthermore, such refinement will not eliminate the need to develop the high-confidence toxicological information that is required to support the predicted RfD.

Confidence in our ability to accurately predict RfDs is based largely upon the fact that our QSAR model’s estimated RfDs are consistent across multiple randomly identified training sets and the precision and accuracy demonstrated through a comparison of compound-specific predicted RfDs and published RfDs. Our confidence is further heightened by noting that the majority (79%) of our predicted RfDs (i.e., the geometric means of four estimated RfDs) are within the accepted order of magnitude (10-fold) of accepted precision and accuracy expected of published RfDs.

Our QSAR model is not without limitations. While our QSAR model demonstrates consistency in predicted RfDs for many of the constituents comprising TPH fractions, our predicted RfDs for constituents of the Aliphatic High Carbon fraction appeared to deviate by two orders of magnitude from the surrogate RfD for white mineral oil used in the risk assessment to assess the human health risk posed by this aliphatic fraction. We suspect that the difference between this surrogate RfD and our predicted RfDs for constituents in the aliphatic fraction are largely due to the surrogate RfD being based on the lowest therapeutic dose and not the absorbed fraction of the therapeutic dose (2%). If the surrogate RfD for the Aliphatic High Carbon fraction were corrected, it would reflect a nearly 100-fold decrease in this fraction’s surrogate RfD (i.e., more toxic). Our current predicted RfDs for constituents of this aliphatic fraction would be much closer to this corrected surrogate RfD than they are at present. Our QSAR model output (i.e., predicted RfDs) also lacks consistency in the domain of the smaller aliphatic compounds with published RfDs (Figure 4). Our predicted RfDs appear to be much less toxic than the surrogate RfD, but are comparable to more than half of the specific compound RfDs used to assess the human health risk posed by this aliphatic fraction. Perhaps more interesting is the apparent range, spanning three orders of magnitude, for both published and predicted RfDs for this fraction. Interestingly, our QSAR model 3 estimate appears to closely mirror published RfDs. This is clearly a result of the randomly selected training set used in QSAR model 3 and the resulting model identification of relevant 2D MDs. For these reasons, and the lack of precision in the predicted RfDs, risk assessors should not rely on the predicted RfDs for this aliphatic fraction without careful consideration of these limitations. Because of the range of predicted and published RfDs for constituents in this fraction, we are unable to define an inflection point for discerning what is outside the applicability domain for this fraction.

In summary, for a compound where the range of QSAR model compound-specific RfD estimates is less than 10-fold, we confidently recommend the use of the resulting predicted RfD in the assessment of human health risk. This includes nearly 80% of the target PIANO analytes (i.e., <8-fold). For a compound where the range of RfD estimates exceeds 10-fold but is less than 20-fold, the predicted RfD might be used with caution. For a compound where the range of QSAR model RfD estimates exceeds 20-fold, risk assessors should not blindly rely on the predicted RfD as a toxicity value of sufficient precision and accuracy. Reviewing the relative toxicity of such compounds, comparing predicted RfDs with published RfDs identified within the applicable TPH fraction might be used by risk assessors to justify the preferential use of a predicted RfD over a surrogate RfD in the assessment of human health risk.

#### 4.2.2. Comparison of Predicted RfD Values with TTC Values

In the absence of chemical-specific toxicity data, Threshold for Toxicological Concern (TTC) values have been used to estimate safe human exposure levels (e.g., RfDs). Regulatory bodies worldwide have employed TTC values to assess the toxicologic potential of hundreds of cosmetic ingredients, fragrance materials, and food additives. Briefly, the TTC approach assigns organic chemicals to one of three classes by applying structural information to the Cramer decision tree or some modification of that decision tree [27]. Each of these classes is associated with a limited daily exposure that is considered safe, reported in units of µg/kg bw/day (1 μg/kg-day = 0.001 mg/kg-day). The low toxicity limit (Class I compounds) is 30 µg/kg bw/day (0.03 mg/kg-day), the intermediate toxicity limit (Class II compounds) is 9 μg/kg-day (0.009 mg/kg-day), and the high toxicity limit (Class III compounds) is 1.5 μg/kg-day (0.0015 mg/kg-day). Pham et al. [27] compared compound-specific TTC values with published oral RfDs and reported that, on average, published RfD values were about 6-fold higher (less toxic) than the respective compound-specific TTC values, which resulted in a health-protective conservative estimation of safe exposure [27].

Our predicted RfDs provide a higher resolution of compound toxicity than the applicable TTC values applied to three classes of compounds. From a simple Cramer decision tree, straight-chain aliphatic compounds like *n*-Pentane (109-66-0) (Figure 5) and *n*-Hexane (110-54-3) (Figure 4) are assigned to Class I with low toxicity (TTC = 30 μg/kg-day). The published oral RfDs for these compounds are 0.286 and 0.06 mg/kg-day, respectively. The TTC values for *n*-Pentane (109-66-0) and *n*-Hexane (110-54-3) Class I TTC compounds are about 9.5-fold and 2-fold lower than the published RfDs, respectively. In contrast, our predicted RfDs for these aliphatics align more closely with the published RfD values, which are about 5.3- to 1.1-fold lower than our predicted RfDs (both are 0.054 mg/kg-day). While TTC values appear to provide conservative, health-protective exposure limits for these compounds, they are significantly lower (more toxic) than their published RfDs and would likely require additional remedial action than would be required of RfDs defining lower toxicity. In contrast, our predicted RfDs for these compounds utilize all the relevant 2D MDs for these compounds to predict RfDs that are within 6-fold of their published RfDs.

To test the relative impact of our predicted RfDs on human health risk assessment, we conducted an assessment of human health risk at a hypothetical site where JP-5 jet fuel was released into drinking water. We assumed that people were only exposed to JP-5 in drinking water through its consumption and that all of the JP-5 target PIANO analytes were detected in contaminated drinking water. We conducted two different assessments of the human health risk. In the first assessment, we used surrogate RfDs for each of the aliphatic and aromatic fractions of JP-5 present in PIANO analyses to assess the human health risk posed by constituents that did not have published RfDs. In a second assessment, we used our predicted RfDs for constituents of JP-5 that did not have published RfDs. In both assessments, we used the published RfDs for all JP-5 constituents that have published RfDs. We found that the second assessment, which used our predicted RfDs, resulted in a 30% lower estimate of human health risk than that determined using surrogate RfD values for the aliphatic and aromatic fractions. This estimate of health risk is highly dependent on individual and fraction-specific surrogate toxicity estimates (i.e., RfDs) and the concentration of JP-5 constituents in drinking water.

### 4.3. Published RfD Outliers

**Figure 8:**
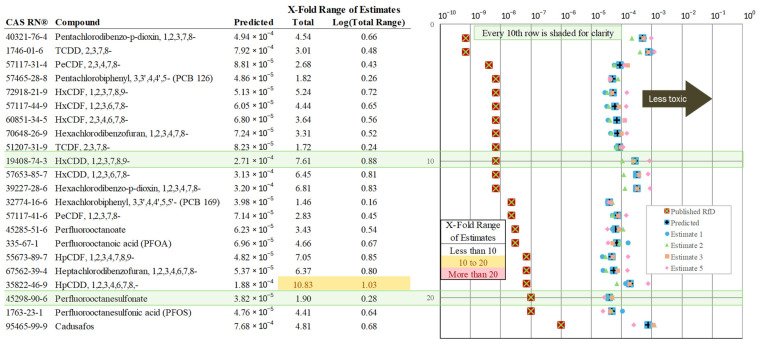
Statistically extreme outliers (mg/kg-day). Comparison of results from four QSAR equations and their geometric means (predicted blue squares) with published RfDs (red squares) of 22 extreme outlier compounds. CAS RN^®^: Chemical Abstracts Service Registry Number, an unambiguous, unique compound identifier.

We identified 22 compounds with published RfDs for elimination from the QSAR model development (Supplementary Table S2). These outliers were more than three standard deviations below the mean of all other published RfDs and were excluded as extreme statistical outliers. Most of these compounds share a history of controversial toxicological assessment, which begs the question: What about these published RfDs is different from those published for other compounds? The excluded compounds (Table 2) included chlorinated dibenzo-p-dioxins and furans, polychlorinated biphenyls 126 and 169, perfluorooctanoic acid (PFOA) (335-67-1), and perfluorooctane sulfonic acid (PFOS) (1763-23-1). These compounds have published RfDs that are two to five orders of magnitude lower (more toxic) than our QSAR model predicted RfDs. For 19 of these compounds, the published RfDs are 1000- to 300,000-fold lower (i.e., more toxic) than our predicted RfDs (Figure 8). Inclusion of the statistical outliers in our QSAR model does not, however, significantly alter how well our predicted compound-specific RfDs match published RfDs. This finding suggests that the 2D MDs associated with these much lower published RfDs do not significantly skew QSAR model performance. This also suggests that the same MDs used to predict RfDs for the outliers do not unduly influence our QSAR model’s reliance on these same MDs for other compound-specific RfD predictions.

Interestingly, the 22 statistical outliers all share a history of significant scientific debate concerning their toxicity assessment. The GAO [13] details how political pushback, interagency disputes, and resource constraints caused toxicity assessments for chemicals like dioxin to drag on for decades. It is not hard to imagine that such factors have “colored” the prescribed approach to RfD derivation for the 22 outlier compounds. This is clearly illustrated by the EPA’s RfDs for PFOA and for PFOS, which are orders of magnitude below those derived by other regulatory bodies and an international consortium of scientific experts. Specifically, the EPA recently revised the RfD for perfluorooctanoic acid (PFOA) (335-67-1) downward to 3.0 × 10^−8^ mg/kg-day [28]. This published RfD is more than three orders of magnitude lower (2320 times more toxic) than our predicted RfD of 6.96 × 10^−5^ mg/kg-day. However, our predicted RfD for PFOA is very near the upper range (1.0 × 10^−5^ to 7.0 × 10^−4^ mg/kg-day) of the recently published safe dose for PFOA (335-67-1) determined by an international collaboration [29]. Similarly, the EPA recently revised the RfD for Perfluorooctane Sulfonic Acid (PFOS) (1763-23-1) downward to 1.0 × 10^−7^ mg/kg-day [30]. This RfD is more than two orders of magnitude lower (476 times more toxic) than our predicted RfD of 4.76 × 10^−5^ mg/kg-day, which lies well within the range (2.0 × 10^−5^ to 1.0 × 10^−4^ mg/kg-day) of a recently published safe dose for PFOS (1763-23-1) determined by another international collaboration [31]. In summary, our QSAR model predicted RfDs for PFOA and PFOS are supported by the published works of two international consortiums, who imply that the EPA’s published RfDs for these compounds do not adhere to applicable RfD derivation guidance or the prescribed process of RfD derivation. Presumably, the EPA used other undocumented information in deriving their published RfDs for PFOA and PFOS [28,30], or were influenced by political pushback, interagency disputes, resource constraints, or other political considerations.

We conducted a sensitivity analysis of our RfD prediction process by including the statistical outliers in our training sets. Generally, the inclusion of statistical outliers did not significantly alter our RfD predictions for PIANO compounds, with our predicted RfDs remaining within the accepted order-of-magnitude precision for published RfDs. Our predicted RfDs for statistical outliers were, as would be expected, closer to their published RfDs (smaller than our original estimates). This effect was most pronounced for dioxins and dibenzofurans, and less so for PCBs and PFASs.

The range of the QSAR model RfD estimates for high-carbon (C31–C40), non-branching aliphatics is larger, but our RfD predictions follow a regular pattern (Figure 5). This may reflect the fact that only four compounds in this fraction have published RfDs and that these do not follow a linear pattern. The published RfDs for the aliphatic compounds n-Pentane (C5) (109-66-0) at 2.86 × 10^−1^, n-Hexane (C6) (110-54-3) at 6.0 × 10^−2^, n-Heptane (C7) (142-85-5) 3.0 × 10^−4^, and n-Nonane (C9) (111-84-2) at 3.0 × 10^−4^, as well as other longer unbranched aliphatic compounds, vary significantly from our predicted RfDs and therefore warrant additional investigation. Finally, as discussed in the Discussion section, the difference between our QSAR model predicted RfDs for constituents in the Aliphatic High Carbon fraction and the surrogate RfD for white mineral oil may be closer than indicated because of a flaw in the way the white mineral oil RfD was calculated.

### 4.4. Future Directions

Future research should focus on the application and use of QSAR models like ours to minimize uncertainty in the use of toxicity values in human health risk assessment and to support more accurate regulatory decisions. In this regard, research efforts should validate the accuracy, precision, and reliability of in silico RfD derivation processes and applicable QSAR model output. Validation of such models should include further assessment of whether the predicted RfDs are supported by compound-specific in vitro and in vivo toxicity assessments. Such assessments might be focused on specific toxicological assessments and prioritized based on our QSAR model’s predicted compound-specific RfDs and the perceived need for regulatory action.

For compounds with published RfDs that are more than an order of magnitude different from our predicted RfDs, further research is warranted. While our QSAR model does not consider the selected critical effect or the application of UFs, it does use the published RfDs and 2D MDs to generate estimated and predicted RfDs. The actual toxicity may be much less or much greater than that predicted by our QSAR model. On the other hand, our QSAR model may signal the need to re-evaluate published RfDs to ensure that the appropriate critical effect is selected and that the level of uncertainty used in the RfD derivation is reasonably estimated. For example, toxicity values for such compounds might benefit from additional epidemiological, biological, and toxicological research. Examples include Triacetin (102-76-1), Cyclohexane (110-82-7), 1,1′-Biphenyl (92-52-4), Phenol (108-95-2), and MTBE (1634-04-4). This is important for two reasons: Where a compound’s published RfD is greater (less toxic than the QSAR-predicted RfD), adopting the predicted RfD will offer greater health protection. Alternatively, where a compound’s published RfD is smaller (more toxic than the QSAR-predicted RfD), adopting the predicted RfD may be hazardous to exposed persons. Such compounds might be prioritized for further evaluation of the toxicological information from which the published RfD is derived.

Our approach to in silico prediction of RfDs can be applied to compounds that lack toxicological information. Future research efforts should focus on providing support for QSAR RfD estimates and validating in silico approaches like ours for predicting RfDs. This has the obvious advantage of limiting animal use in toxicological research while prioritizing additional toxicological research activities. For example, future QSAR models might be developed that use, in addition to published RfDs, specific toxicological information like the mechanism of action (MOA), tissues affected, uncertainty factor application, 2- or 3D MDs, and other RfD derivation-specific parameters to predict compound-specific RfDs. Such in silico approaches may also be useful in evaluating the EPA process for deriving RfDs. For example, while Figure 5 shows a relationship between increasing toxicity and increasing carbon numbers of linear aliphatics, the actual toxicity may not reflect their gastrointestinal and/or dermal absorption.

Where our QSAR-predicted RfDs differ substantially from published RfDs, the need for additional toxicological assessment is suggested. Such assessment might rely on New Approach Methods (NAMSs) that use novel in vitro test methods to assess whole-system toxicity in meaningful ways. Future in silico research may use 3D descriptors and machine learning approaches. While 3D descriptors are preferred for predicting quantum mechanics properties (e.g., docking of chemicals), both 2- and 3D descriptors are reported to be effective for predicting the activity of small molecules against specific biologicals [32].

### 4.5. Limitations

Our QSAR model was trained using published RfDs and 2D molecular descriptors from recognized authoritative sources. Published RfDs are derived according to the process outlined in the U.S. EPA guidance [6] and verified before being published in the U.S. EPA’s Integrated Risk Information System (IRIS) or Oak Ridge National Laboratories Risk Assessment Information System (RAIS). Because of this, the use of published RfDs as the endpoint of interest in QSAR modeling tacitly includes consideration of all derivation process steps and the available toxicological information used in RfD derivation, including the selection of the critical toxicological effect and the application of Uncertainty Factors (UFs). While the process of deriving compound-specific RfDs includes significant professional judgement, the fact that the majority of our predicted chemical-specific RfDs are within an order of magnitude of published RfDs is proof that the EPA’s RfD derivation process works well even when the available toxicological information is incomplete and professional judgement is used to justify the selection and use of UFs.

We identified 22 outliers as having predicted RfDs that are very different from their published RfDs. These outliers reflect only a small fraction of the total number of compounds for which we predicted RfDs (0.3%). Their inclusion within our QSAR models does not significantly alter our results. The presence of these outliers suggests, however, a limitation to our QSAR model that warrants further discussion. We suspect that the derivation of published RfDs for outliers included “considerations” that were not identified in applicable guidance. These “considerations” appear to have been largely policy-based, as the outliers identified have a controversial history of RfD development or do not follow the derivation process prescribed in the applicable guidance (e.g., Triacetin, 102-76-1). We do not expect the difference between predicted and published RfDs to be a result of errors in either the selection of the critical effect or in the use of UFs. This is because UFs are applied to account for uncertainty in several areas of RfD derivation, including whether or not the existing toxicological database includes information on potential critical effects that might have been identified if the compound of interest had been studied more completely (e.g., the toxicological information does not include an appropriate 2-year reproduction study). The fact that the majority of our predicted RfDs are within an order of magnitude of published RfDs indicates that the use of UFs appropriately addresses this uncertainty, which is inherent in the use of limited toxicological information in RfD derivation. We trust that the majority of the published RfDs were derived by adhering to the process described in the U.S. EPA guidance. For the vast majority of published RfDs, that trust is not misplaced except where we have identified outlier compounds.

## 5. Conclusions

We predict RfDs for 294 PIANO compounds with the accepted level of precision for RfDs. Our approach to in silico prediction of RfDs results in less uncertainty than the use of surrogate compound RfDs in human health risk assessment. Our in silico process for deriving compound-specific RfDs is generally applicable to all organic compounds. Future research efforts should focus on providing support for and validating QSAR RfD estimates for use in risk assessment and in chemical regulatory actions.

## Figures and Tables

**Table 1:** EPA surrogate toxicity values. Fractions are sequenced by primary fractions in alphabetical order and secondary fractions by increasing carbon counts.

Primary Fractions	Secondary Fractions ^a^	Surrogate RfDs (mg/kg-day)	Toxicological Basis
Aliphatics	Low carbon range (C5–C8)	5.0 × 10^−3^	Cyclohexene
	Medium carbon range (C9–C18)	1.0 × 10^−2^	Mid-range aliphatic streams (<1% aromatics)
	High carbon range (C19–C32)	3.0 × 10^0^	White mineral oil
Aromatics	Low carbon range (C6–C8)	Assessed individually	Benzene, ethylbenzene, toluene, xylenes
	Medium carbon range (C9–C16)	1.0 × 10^−2^	Trimethylbenzenes
	High carbon range (C17–C32)	3.0 × 10^−4^	Benzo [a]pyrene

EPA surrogate toxicity values for Total Petroleum Hydrocarbon fractions (mg/kg-day) [12]. ^a^ Carbon ranges identify all compounds found between the respective carbon numbers. For example, the aliphatic high carbon range is composed of all compounds with 19 to 32 carbon atoms in their structure. C: when followed by a number, this symbol represents the number of carbon atoms in the compound; RfD: chronic oral reference dose.

**Table 2:** Compounds with Log(RfD) values that were statistically identified as extreme outliers. CAS RN^®^: Chemical Abstracts Service Registry Number, an unambiguous, unique compound identifier; RfD: chronic oral reference dose.

CAS RN^®^	Compound Name	Published RfD	Log of Published RfD
40321-76-4	1,2,3,7,8-Pentachlorodibenzo-p-dioxin (PCDD)	7.00 × 10^−10^	−9.15
1746-01-6	2,3,7,8-Tetrachlorodibenzo-p-dioxin (TCDD)	7.00 × 10^−10^	−9.15
57117-31-4	2,3,4,7,8-Pentachlorodibenzofuran (PeCDF)	4.00 × 10^−9^	−8.40
70648-26-9	1,2,3,4,7,8-Hexachlorodibenzofuran (HxCDF)	7.00 × 10^−9^	−8.15
39227-28-6	1,2,3,4,7,8-Hexachlorodibenzo-p-dioxin (HxCFD)	7.00 × 10^−9^	−8.15
57653-85-7	1,2,3,6,7,8-Hexachlorodibenzo-p-dioxin (HxCDD)	7.00 × 10^−9^	−8.15
19408-74-3	1,2,3,7,8,9-Hexachlorodibenzo-p-dioxin (HxCDD)	7.00 × 10^−9^	−8.15
57117-44-9	1,2,3,6,7,8-Hexachlorodibenzofuran (HxCDF)	7.00 × 10^−9^	−8.15
72918-21-9	1,2,3,7,8,9-Hexachlorodibenzofuran (HxCDF)	7.00 × 10^−9^	−8.15
60851-34-5	2,3,4,6,7,8-Hexachlorodibenzofuran (HxCDF)	7.00 × 10^−9^	−8.15
57465-28-8	3,3′,4,4′,5-Pentachlorobiphenyl (PCB 126)	7.00 × 10^−9^	−8.15
51207-31-9	2,3,7,8-Tetrachlorodibenzofuran (TCDF)	7.00 × 10^−9^	−8.15
32774-16-6	3,3′,4,4′,5,5′-Hexachlorobiphenyl (PCB 169)	2.33 × 10^−8^	−7.63
57117-41-6	1,2,3,7,8-Pentachlorodibenzofuran (PeCDF)	2.33 × 10^−8^	−7.63
45285-51-6	Perfluorooctanoate (PFOA)	3.00 × 10^−8^	−7.52
335-67-1	Perfluorooctanoic acid (PFOA)	3.00 × 10^−8^	−7.52
67562-39-4	1,2,3,4,6,7,8-Heptachlorodibenzofuran (HpCDF)	7.00 × 10^-8^	−7.15
35822-46-9	1,2,3,4,6,7,8-Heptachlorodibenzo-p-dioxin (HpCDD)	7.00 × 10^-8^	−7.15
55673-89-7	1,2,3,4,7,8,9-Heptachlorodibenzofuran (HpCDF)	7.00 × 10^-8^	−7.15
45298-90-6	Perfluorooctanesulfonate	1.00 × 10^-7^	−7.00
1763-23-1	Perfluorooctanesulfonic acid (PFOS)	1.00 × 10^-7^	−7.00
95465-99-9	Cadusafos	1.00 × 10^-6^	−6.00

**Table 3:** Quantitative performance metrics for the five QSAR models. n: number of compounds in the category; PIANO: the fit statistics when limited to PIANO compounds; Non-PIANO: the fit statistics when applied to non-PIANO compounds; QSAR: Quantitative Structure–Activity Relationship; RMSE: root mean square error; R-square: the coefficient of determination, an indicator of the percentage of variation explained by the model; Adj R-Sq: Adjusted R Square, which accounts for the number of data points and variables in the equation; Q-Square: the coefficient of determination when the equation is applied to compounds not included in the model.

QSAR Model Equation	RMSE	R-Square (n)	Adj R-Sq	Q-Square (n)
1	0.9679	0.5009 (661)	0.4738	0.1799 (183)
2	0.9910	0.4416 (661)	0.4196	0.4128 (183)
3	0.9365	0.4933 (661)	0.4708	0.1899 (183)
4	0.9621	0.4732 (661)	0.4524	0.0593 (183)
5	0.9868	0.4604 (661)	0.4356	0.2423 (183)
PIANO	0.7991	0.5033 (21)	--	0.3513 (22)
Non-PIANO	0.9764	0.4384 (640)	--	0.8408 (161)

## Data Availability

The resources used and the results presented in this study are available in the Supplementary Materials. The SAS Code used for analysis, including instructions, is available upon request from the corresponding author. The EPA’s comprehensive Toxicity Estimation Software Tool database of Molecular Descriptors is available at https://www.epa.gov/chemical-research/toxicity-estimation-software-tool-test (accessed on 24 March 2026). Its user guide can be found at https://www.epa.gov/sites/default/files/2016-05/documents/600r16058.pdf (accessed on 14 June 2026).

## Supplementary Materials

The following supporting information can be downloaded at https://www.mdpi.com/article/10.3390/toxics14060529/s1: Figures S1–S5: Regression Diagnostics for selected models. An example model label of t2e07s07p30 means training set 2, SLE 0.07, SLS 0.07, using variables applicable to at least 30% of compounds; Table S1: Master Set: Identification of Compound-Specific Toxicology Values and Basis for Elimination; Table S2: Betas and RMSEs; Table S3: Descriptors in top models; Table S4: Predicted and Published RfDs; Table S5: Molecular Descriptor values from T.E.S.T.

## Abbreviations

The following abbreviations are used in this manuscript:

BMDL	Benchmark Dose Lower Confidence Limit
CAS RN^®^	Chemical Abstracts Service Registry Numbers^®^
EPA	United States Environmental Protection Agency
IRIS	Integrated Risk Information System
LOAEL	Low Observed Adverse Effect Level
Log(RfD)	The base ten logarithm of the RfD
Mallow’s Cp	A statistically derived value useful in identifying regression models with “appropriate” numbers of variables
MD	2-dimensional molecular descriptors
NOAEL	No Observed Adverse Effect Level
PIANO	Paraffin, Isoparaffin, Aromatic, Naphthene, and Olefin compounds
RAIS	Risk Assessment Information Service
RfC	Chronic inhalation reference concentration
RfD	Non-cancer, chronic oral reference dose
TEST	EPA’s Toxicity Estimation Software Tool
TPH	Total Petroleum Hydrocarbon
2D	2-dimensional
