# Supplementary figures and images for: In Silico Prediction of Chronic Oral Reference Doses for PIANO Target Analytes
Source: Toxics. 2026 Jun 18;14(6):529. doi: 10.3390/toxics14060529 (PMC13308117; doi:10.3390/toxics14060529)

The REG Procedure

Model: t1e07s07p30

Dependent Variable: logRfD

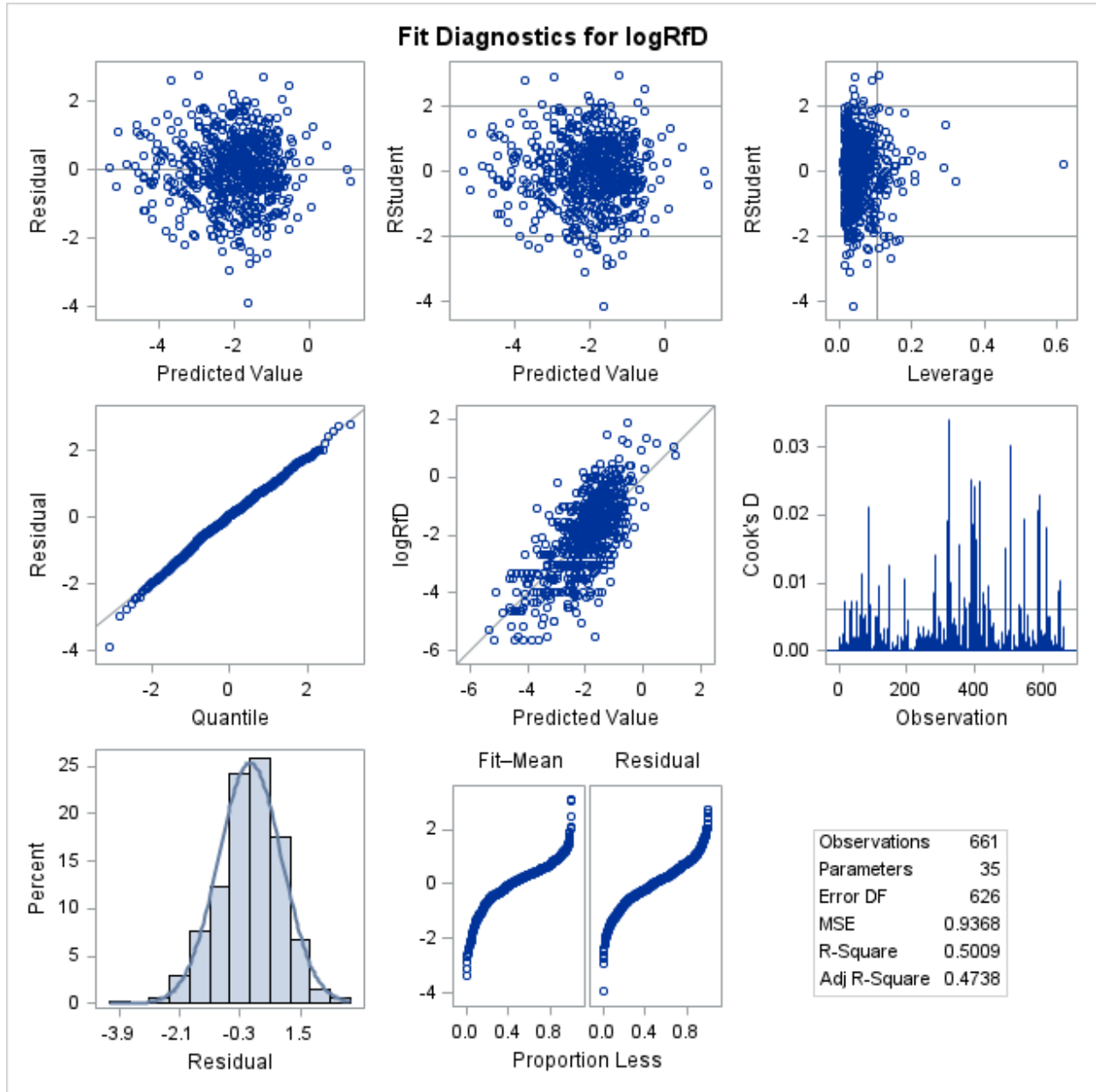

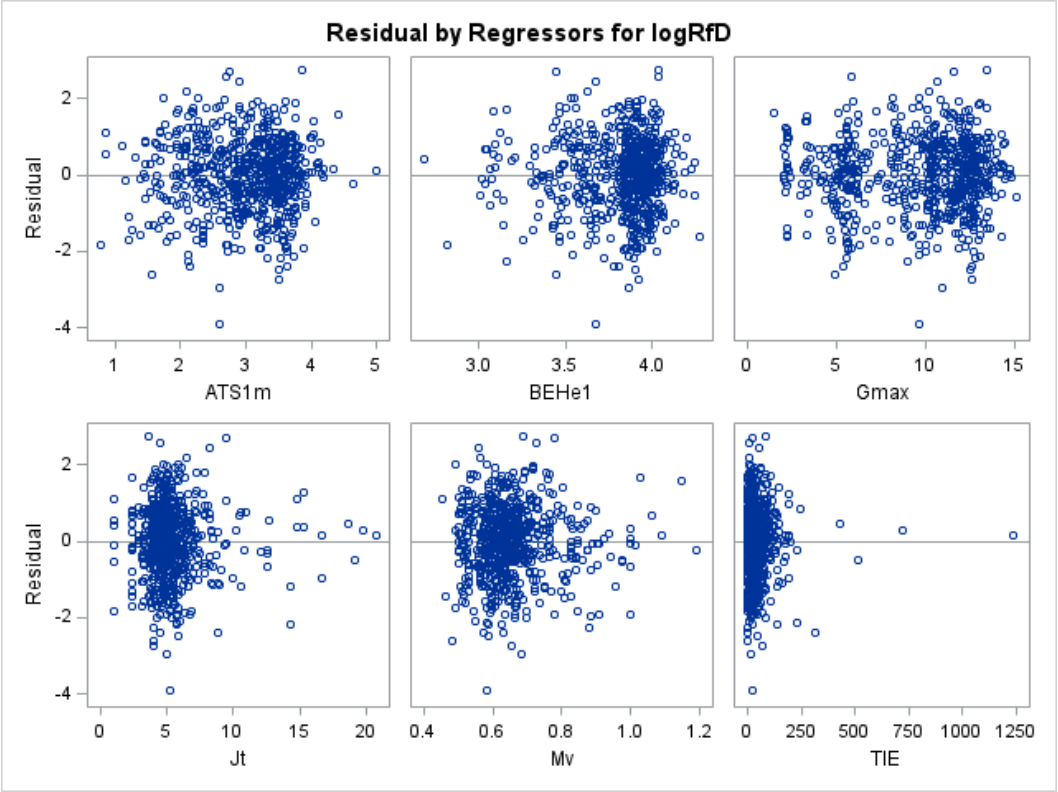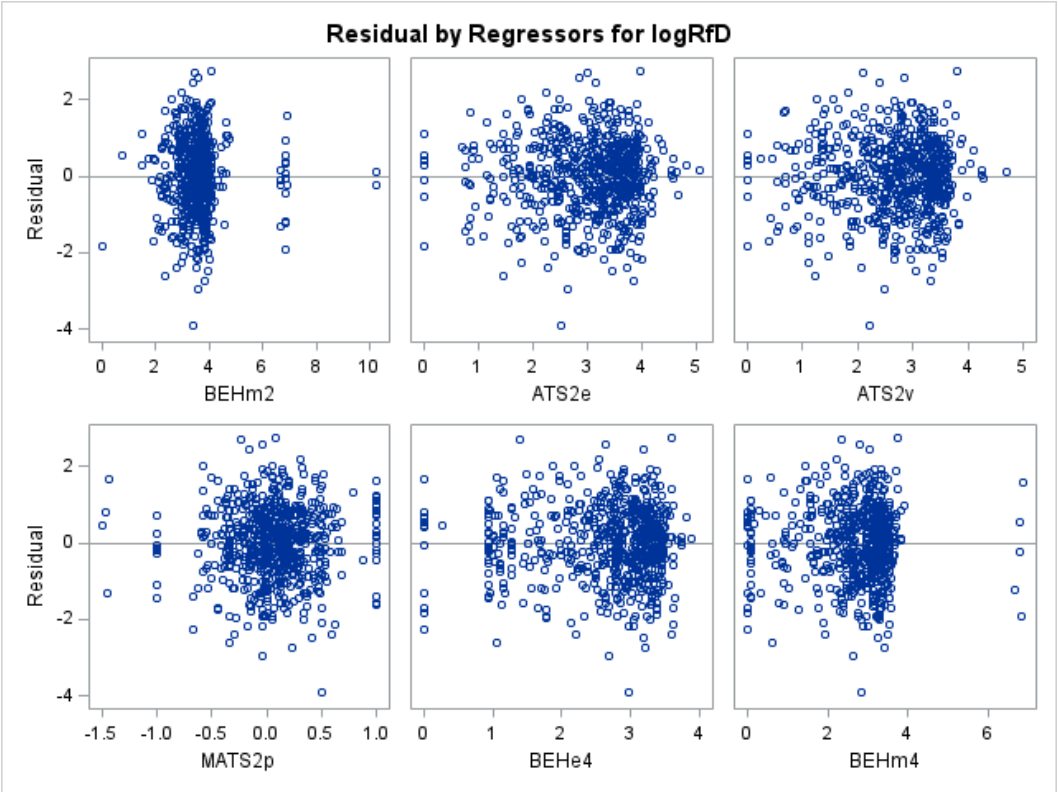

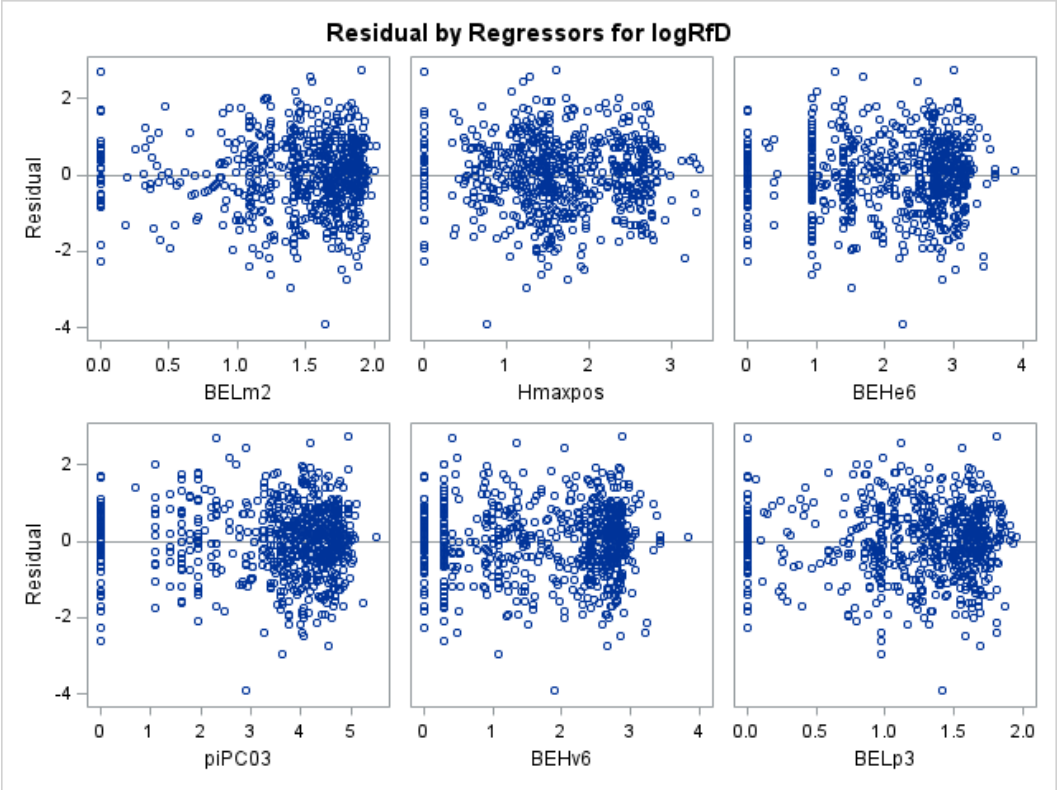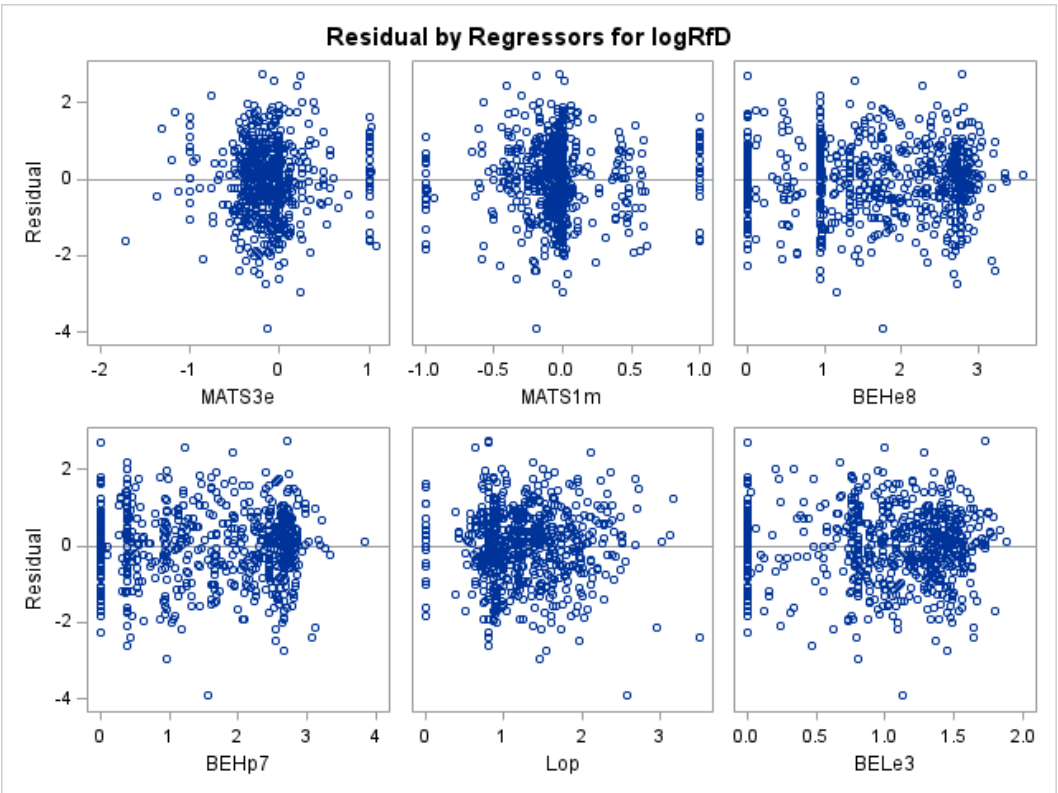

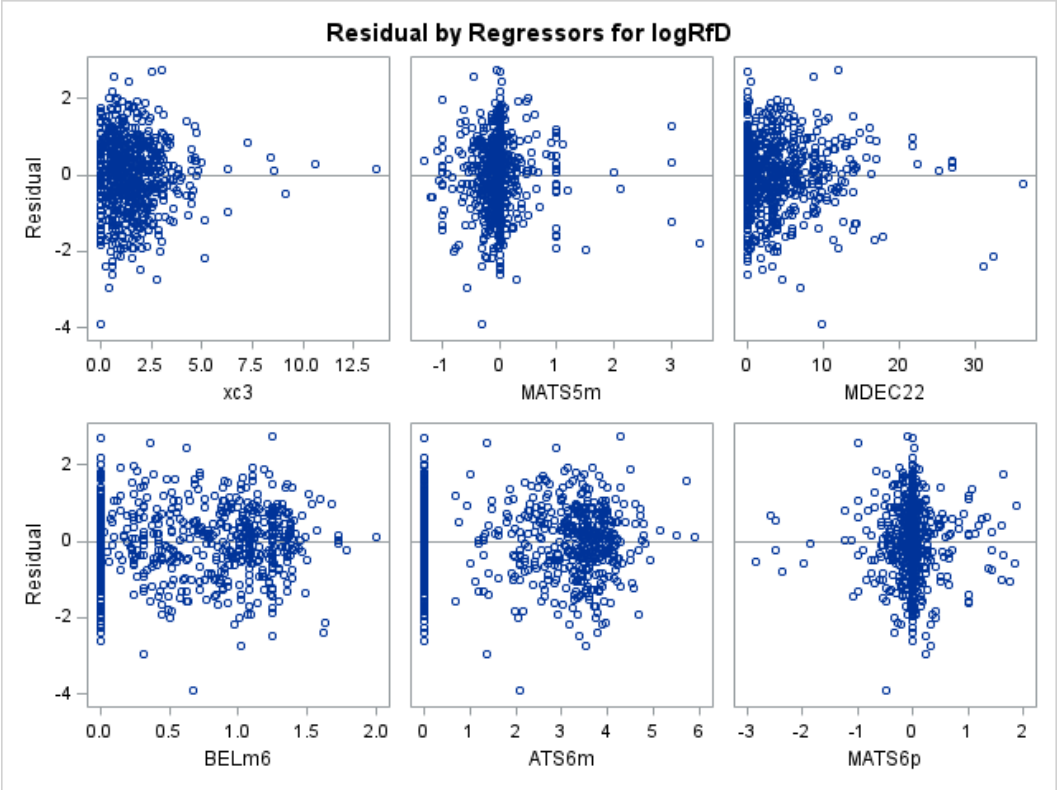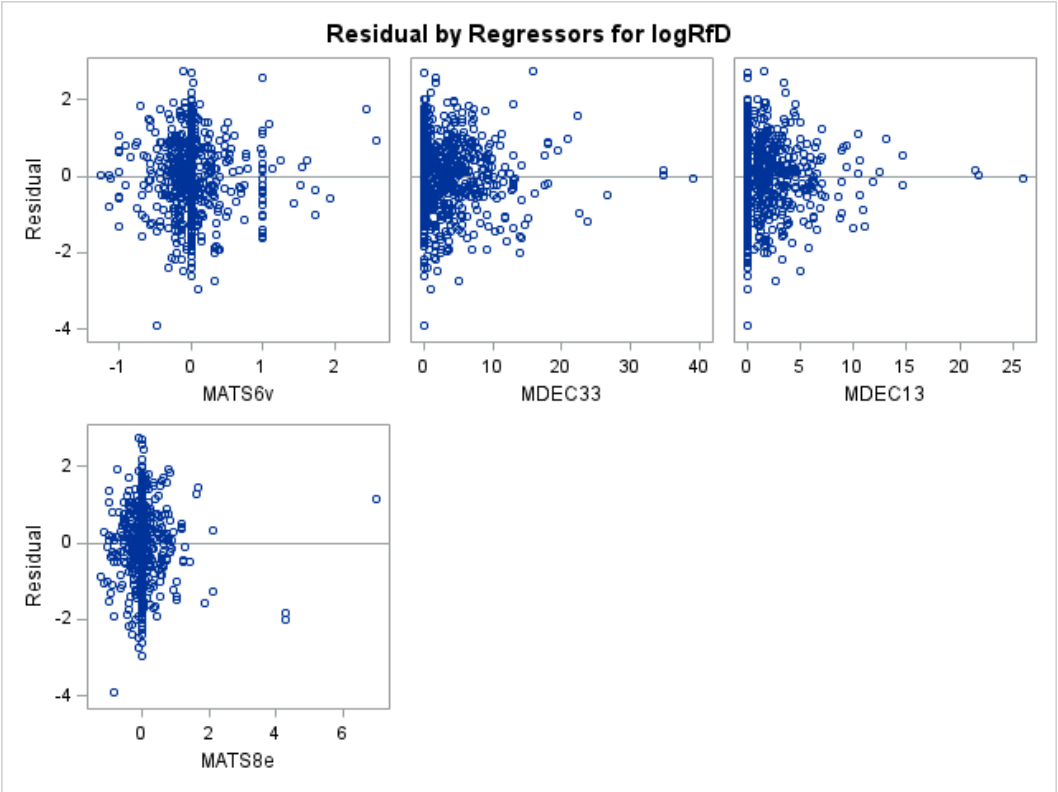

Supplement: Supplementary file 1 [file toxics-14-00529-s001.zip › Supplemental_Graph_Set_t1e07s07p30.pdf]

## The REG Procedure

Model: t2e07s07p30

Dependent Variable: logRfD

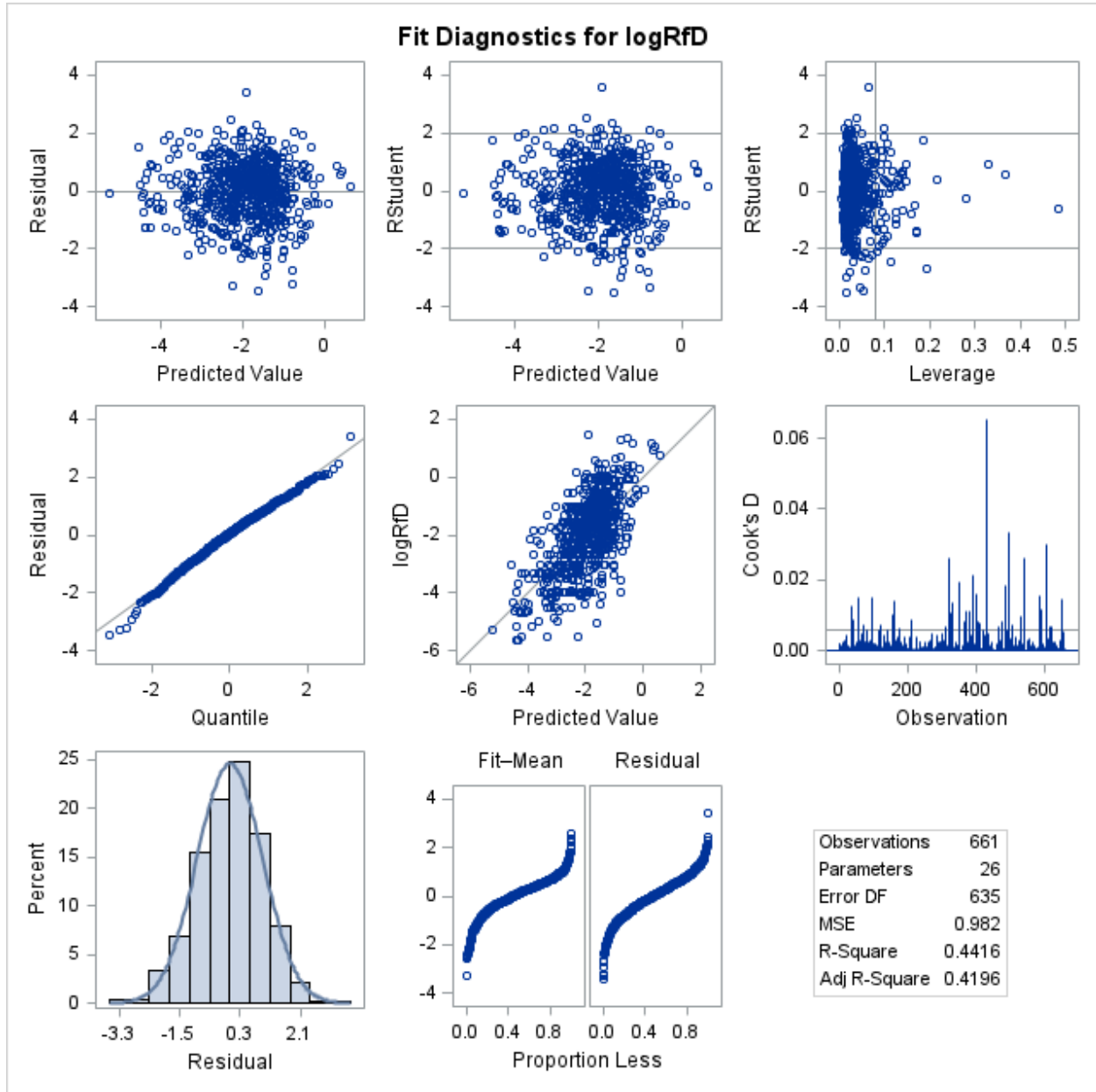

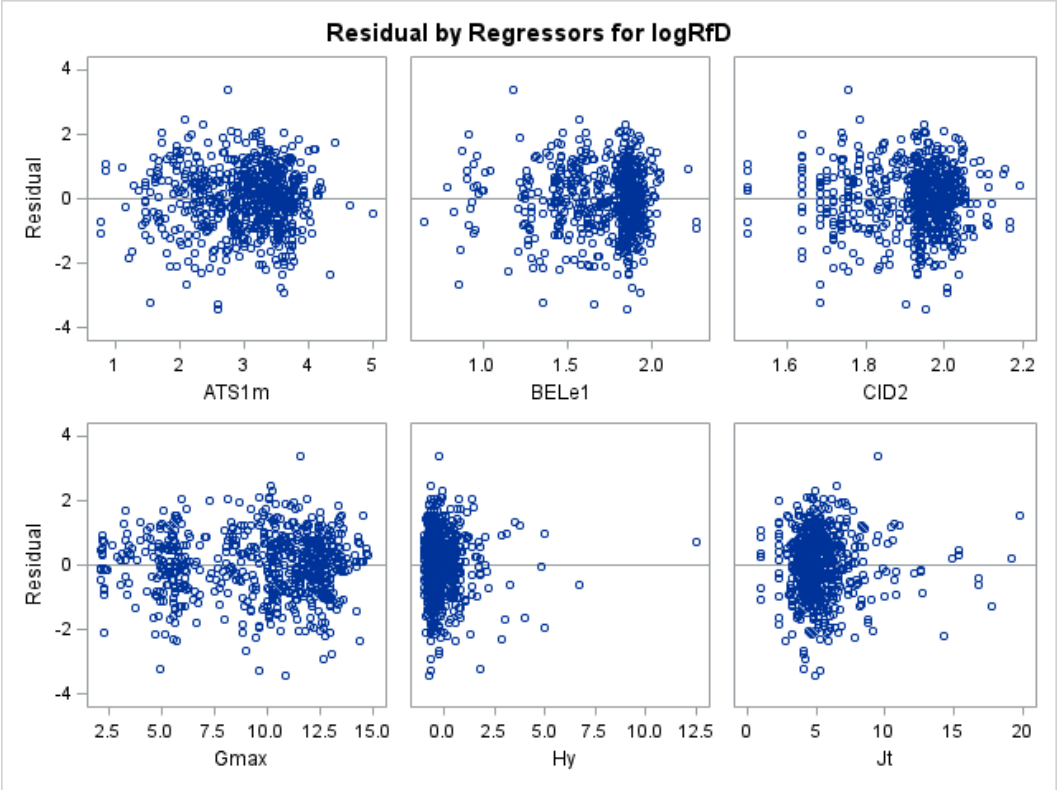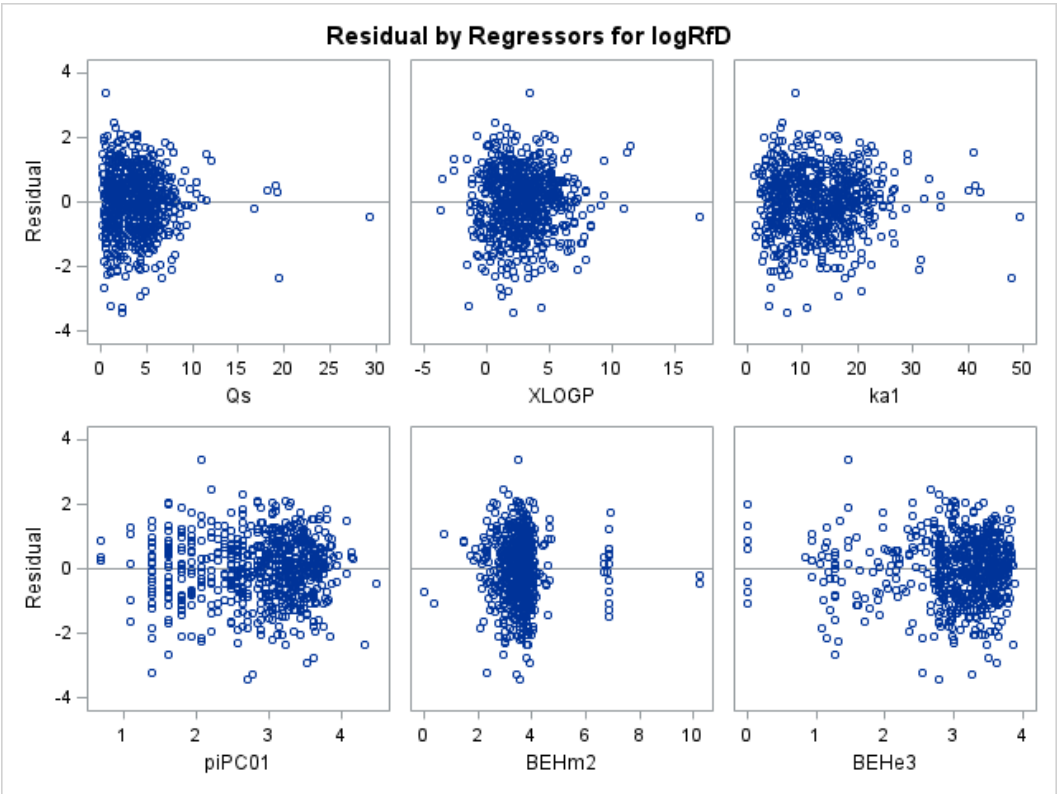

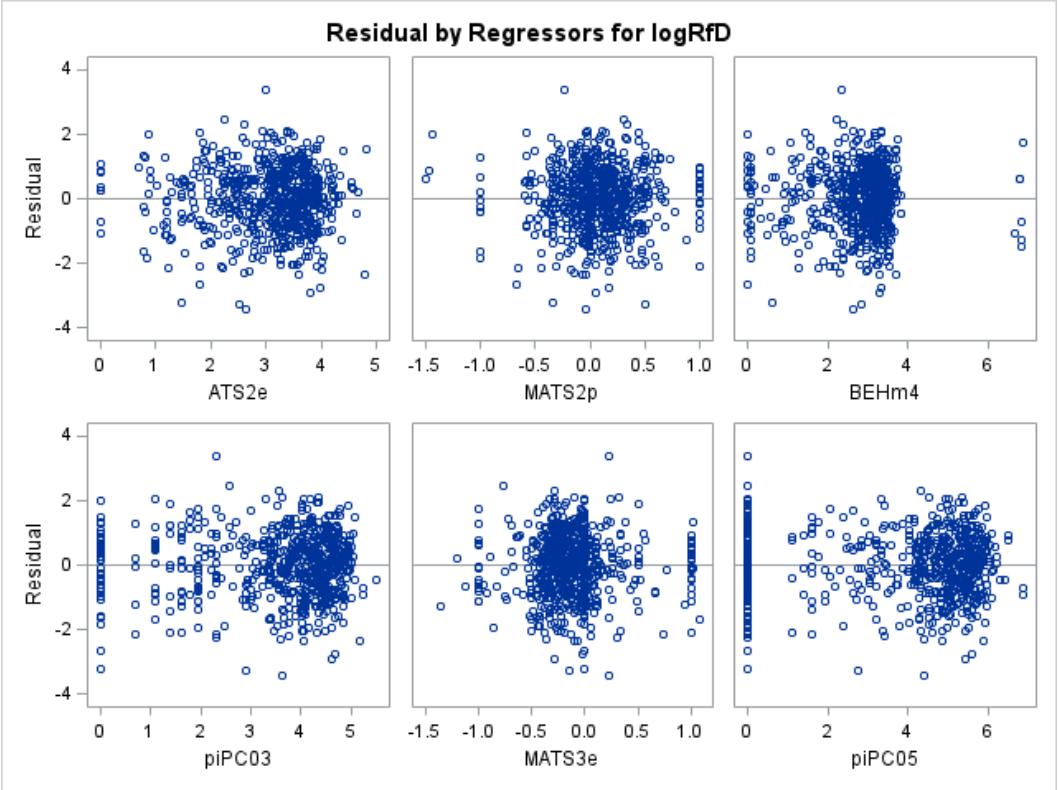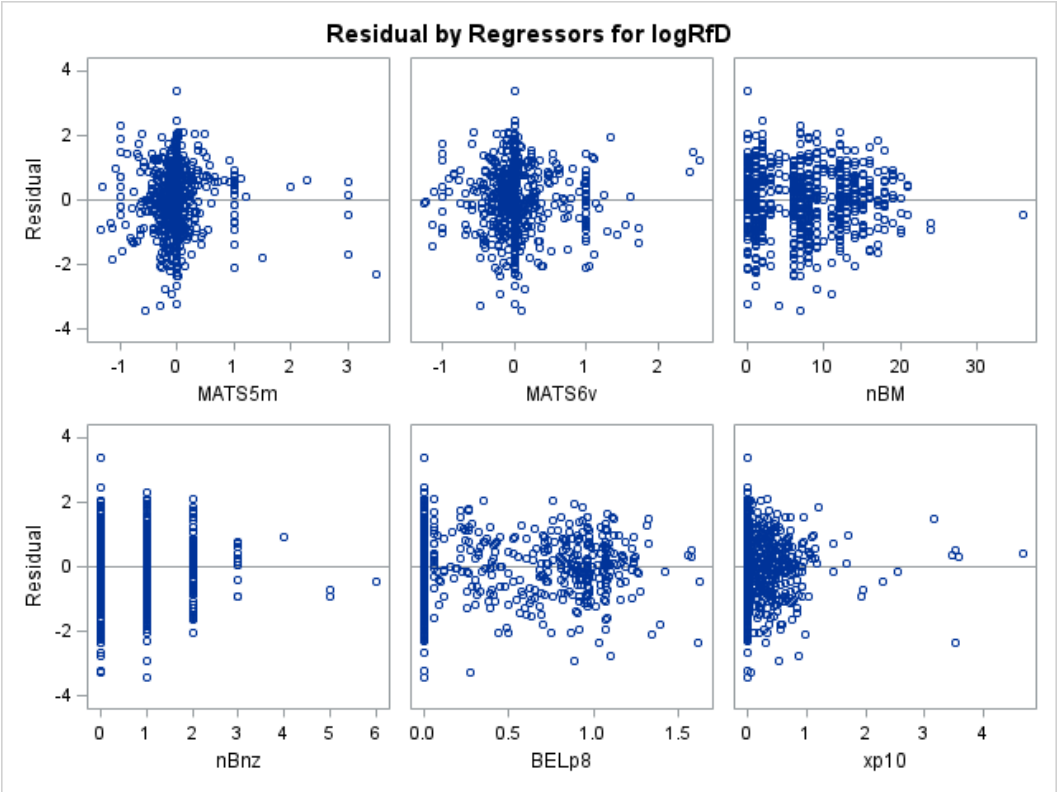

**Residual by Regressors for logRfD**

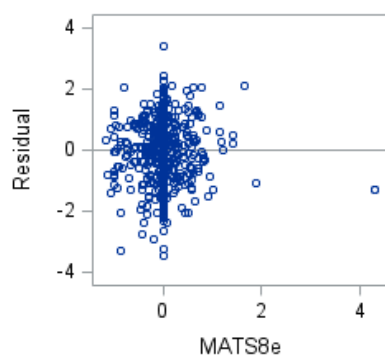

Supplement: Supplementary file 1 [file toxics-14-00529-s001.zip › Supplemental_Graph_Set_t2e07s07p30.pdf]

The REG Procedure

Model: t3e07s07p30

Dependent Variable: logRfD

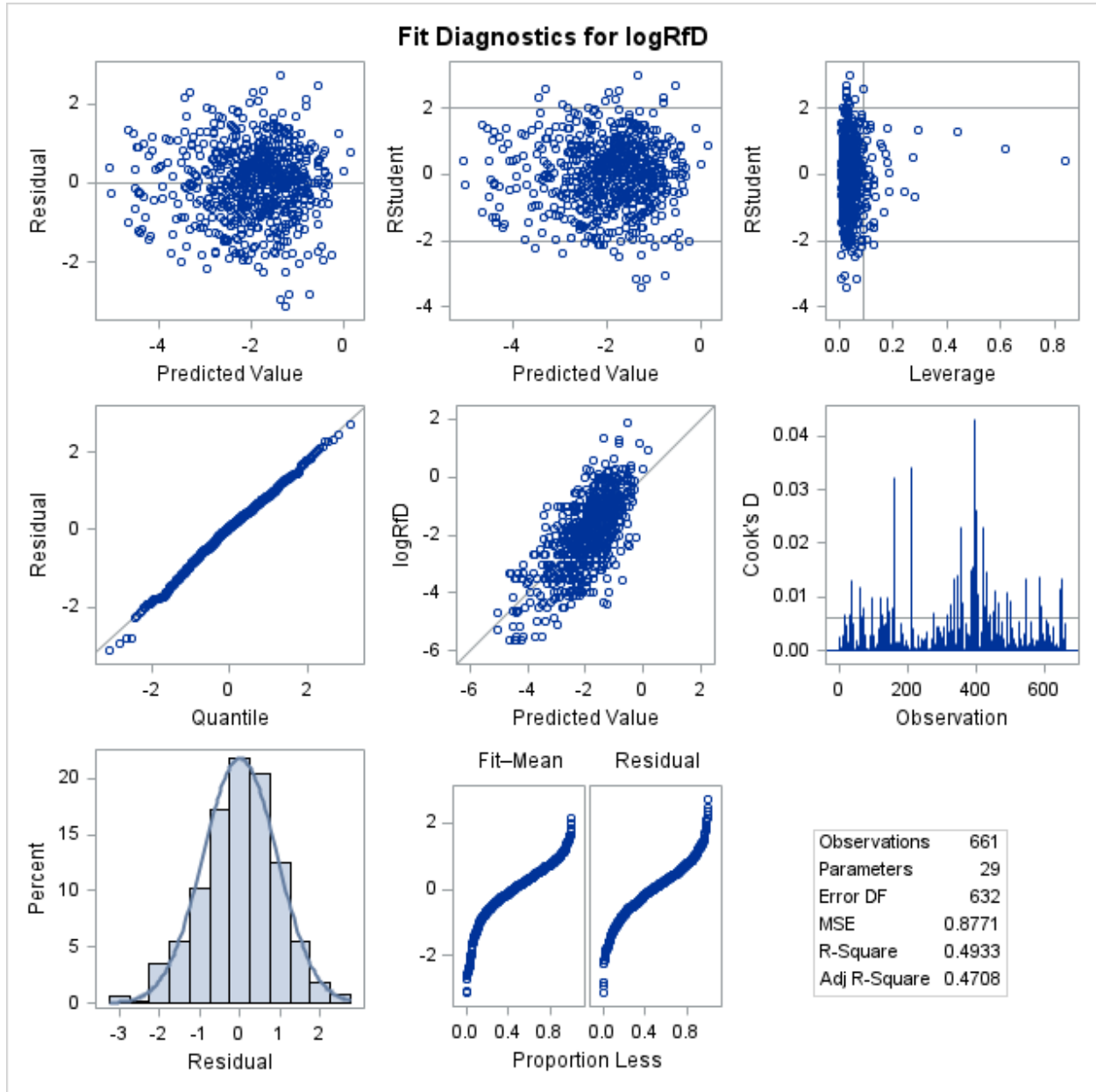

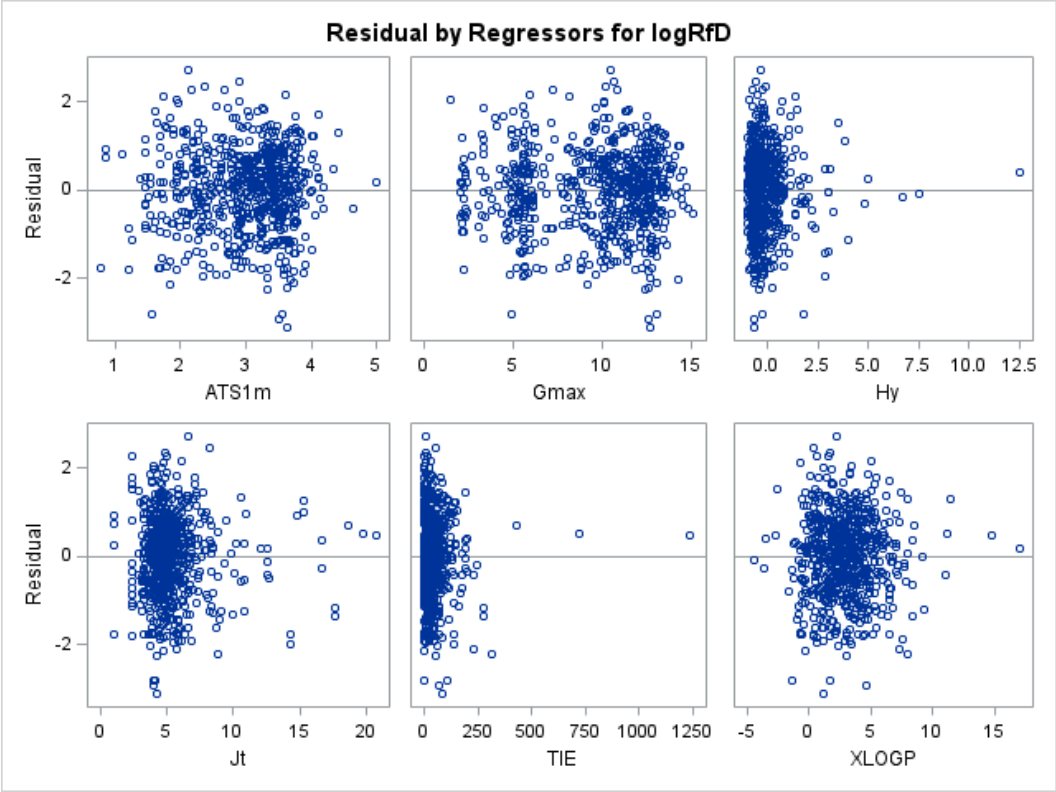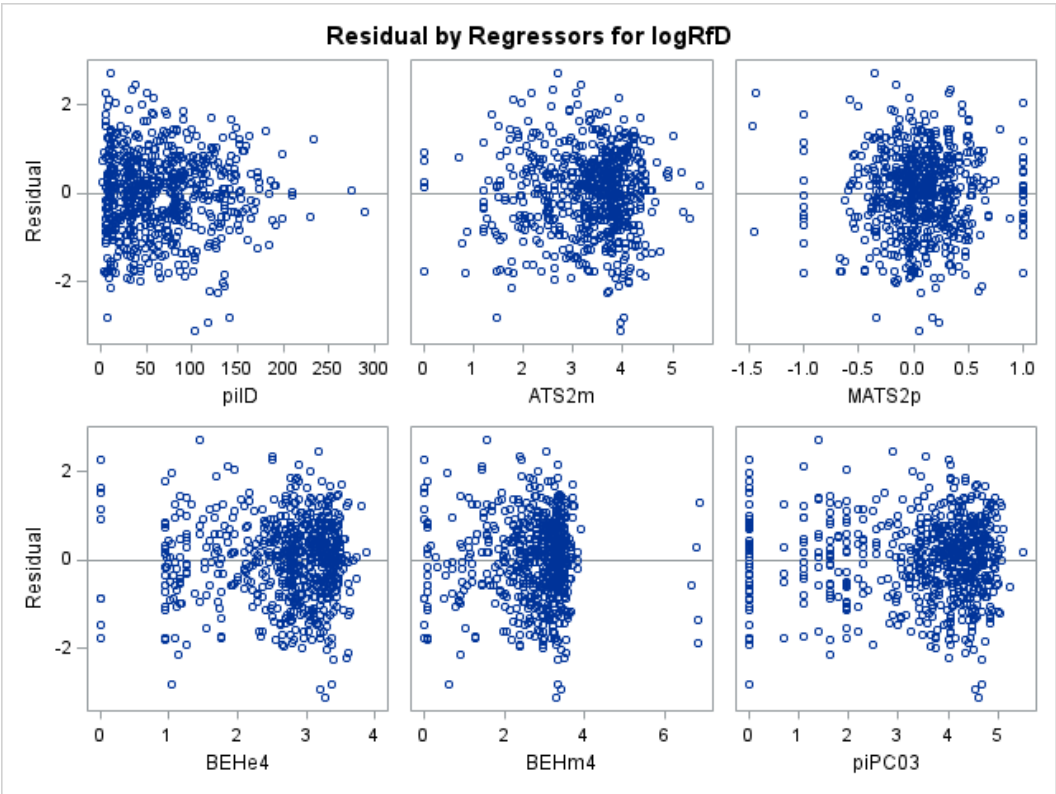

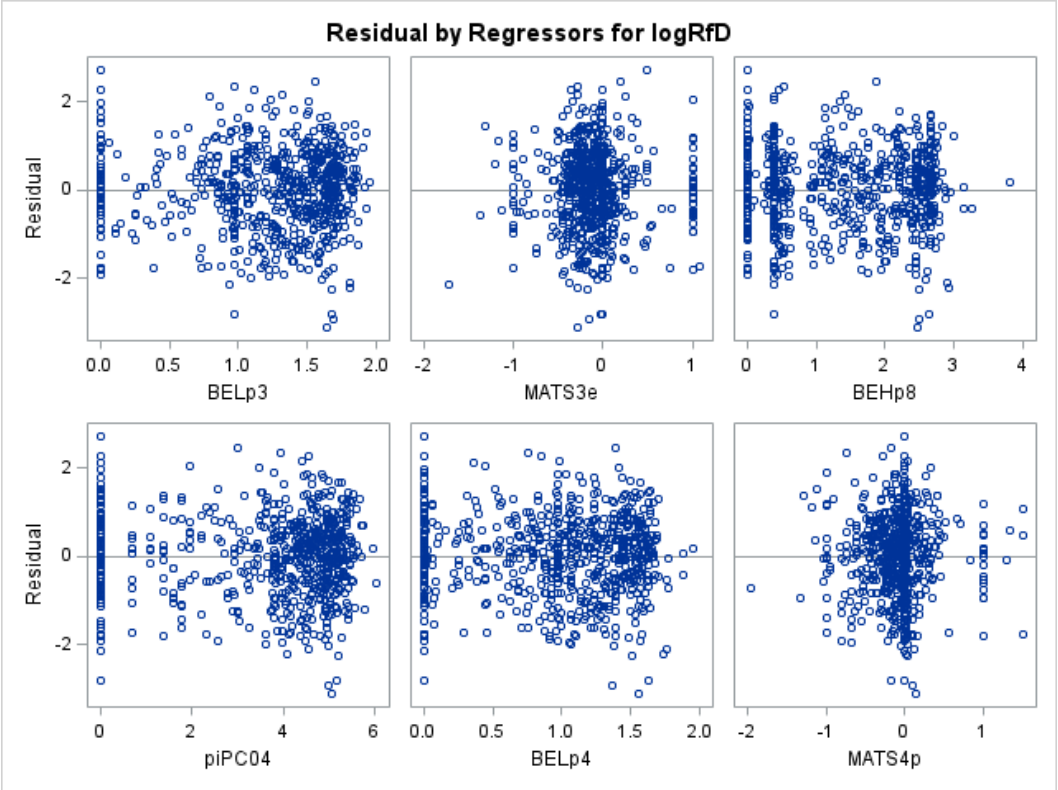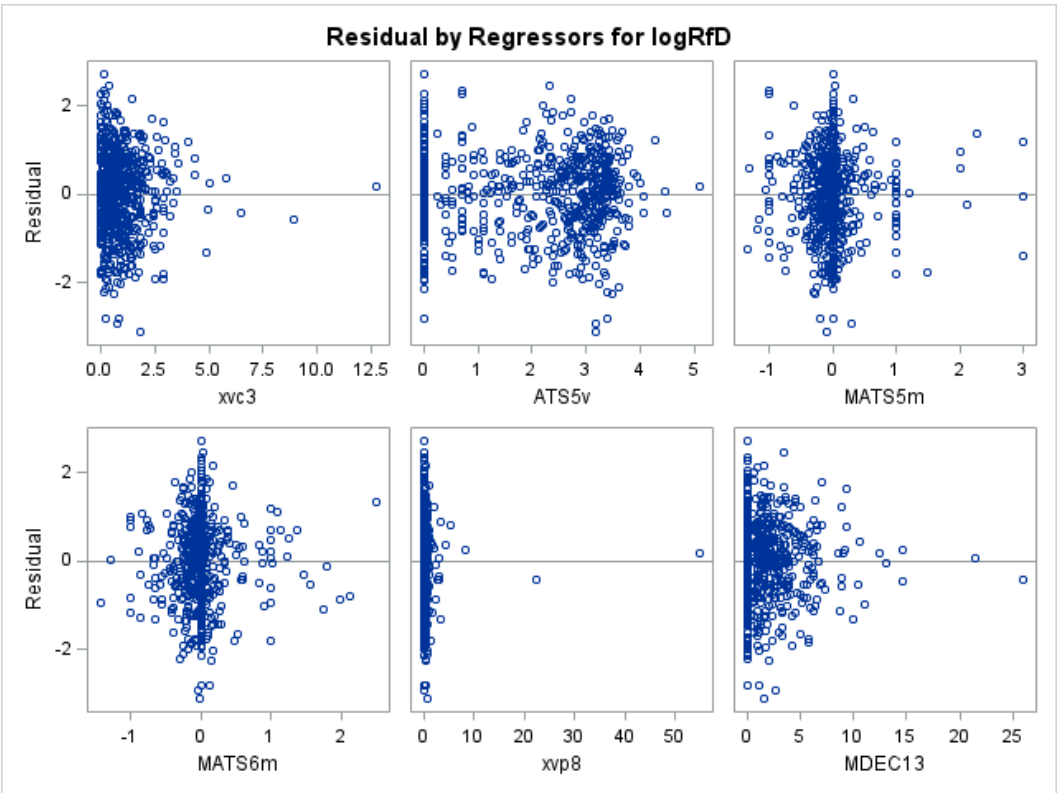

**Residual by Regressors for logRfD**

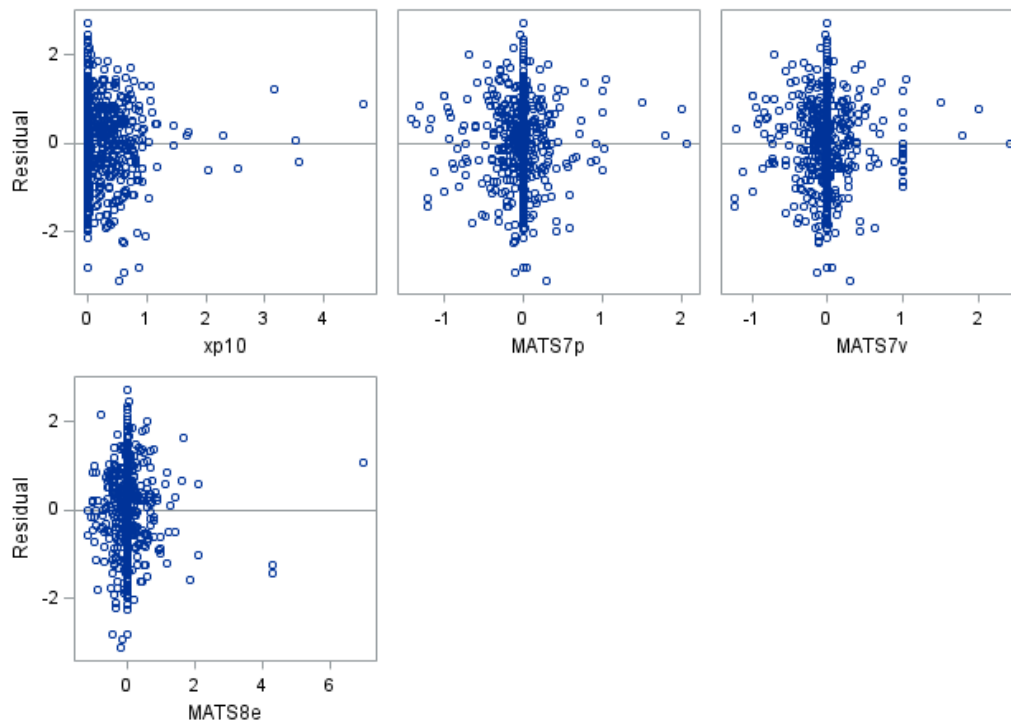

Supplement: Supplementary file 1 [file toxics-14-00529-s001.zip › Supplemental_Graph_Set_t3e07s07p30.pdf]

The REG Procedure

Model: t4e07s07p30

Dependent Variable: logRfD

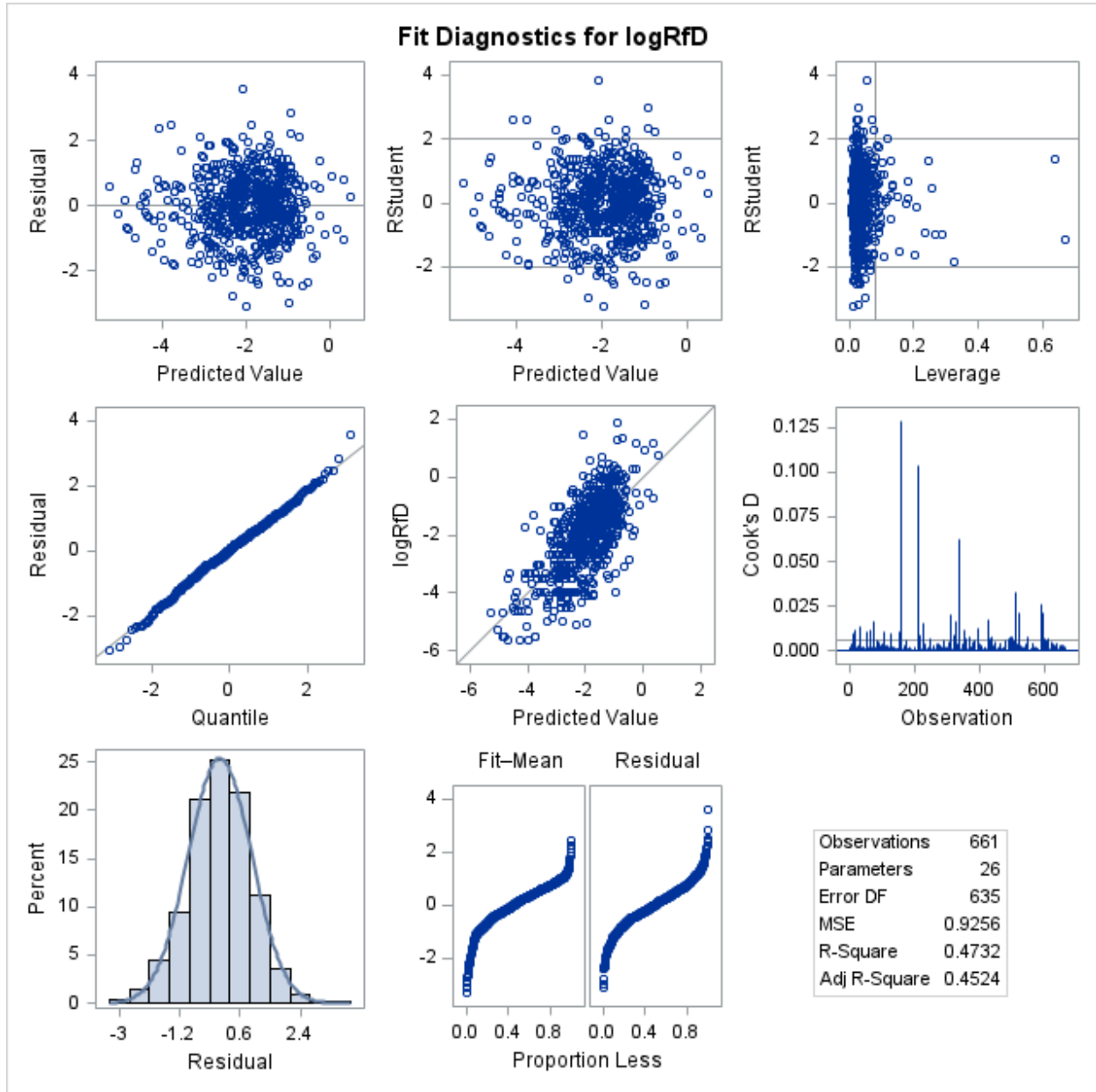

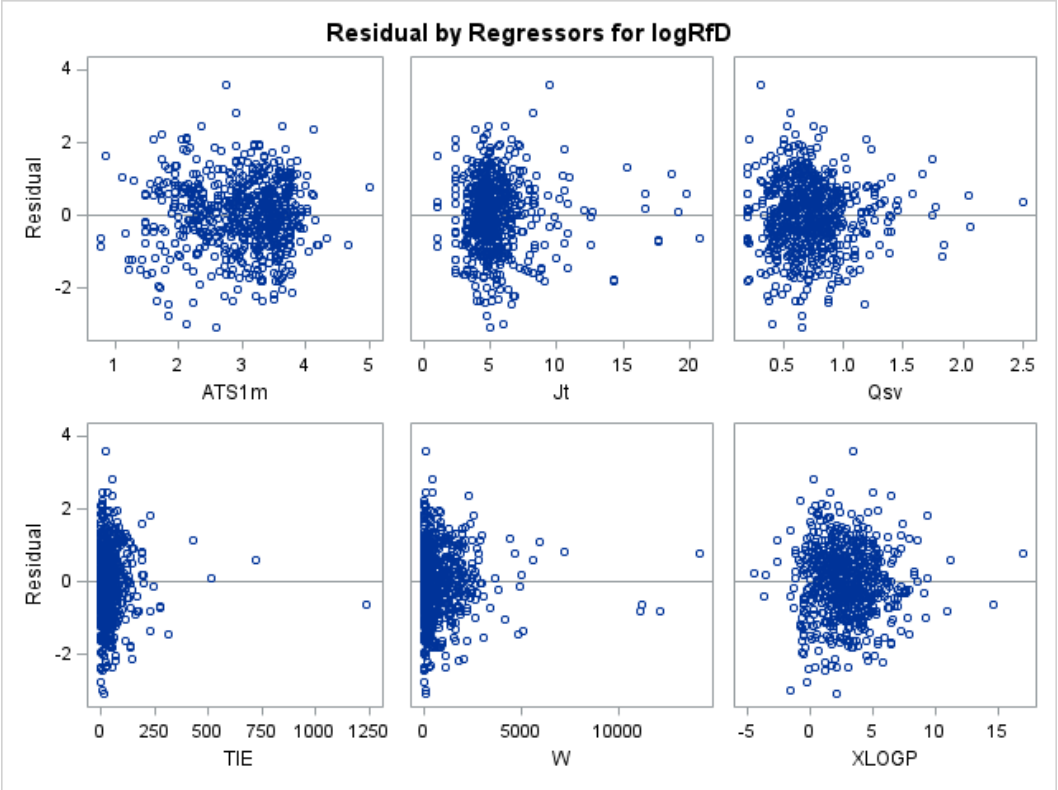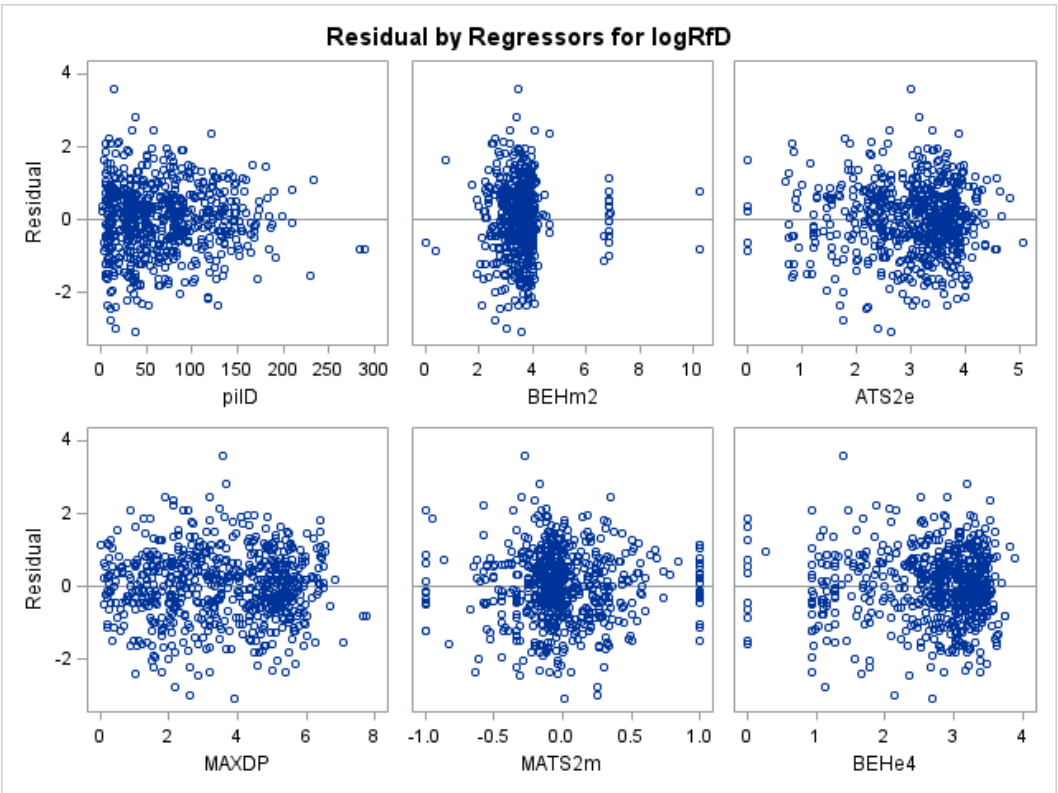

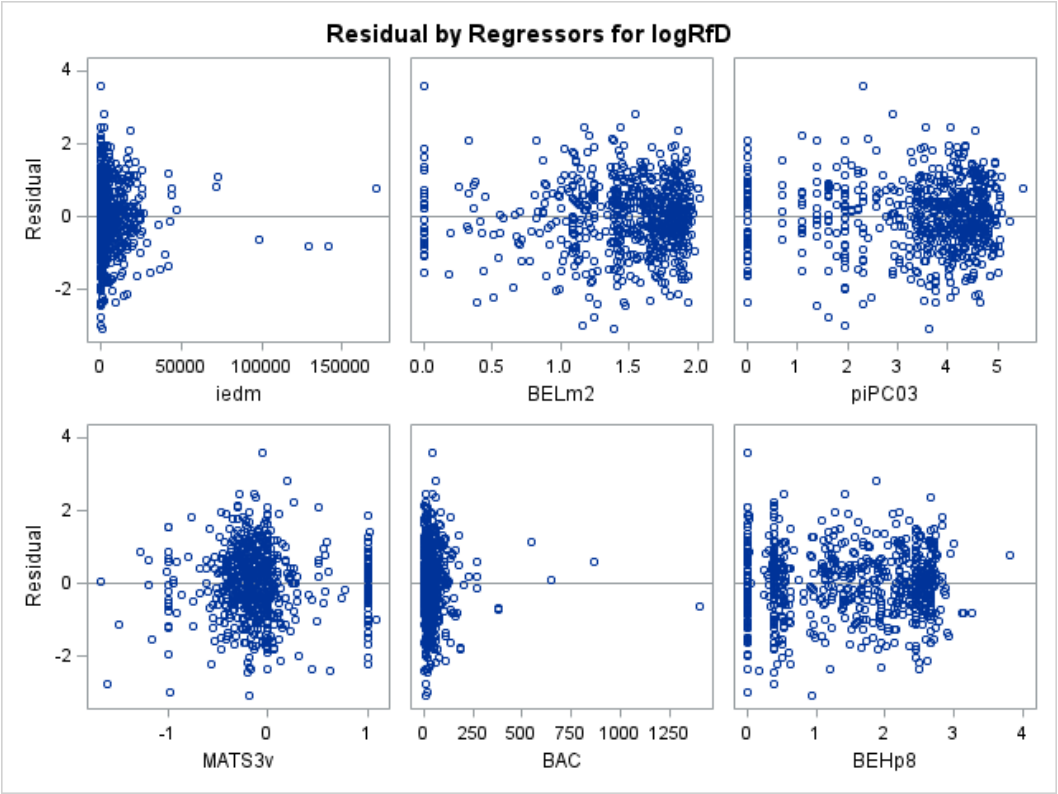

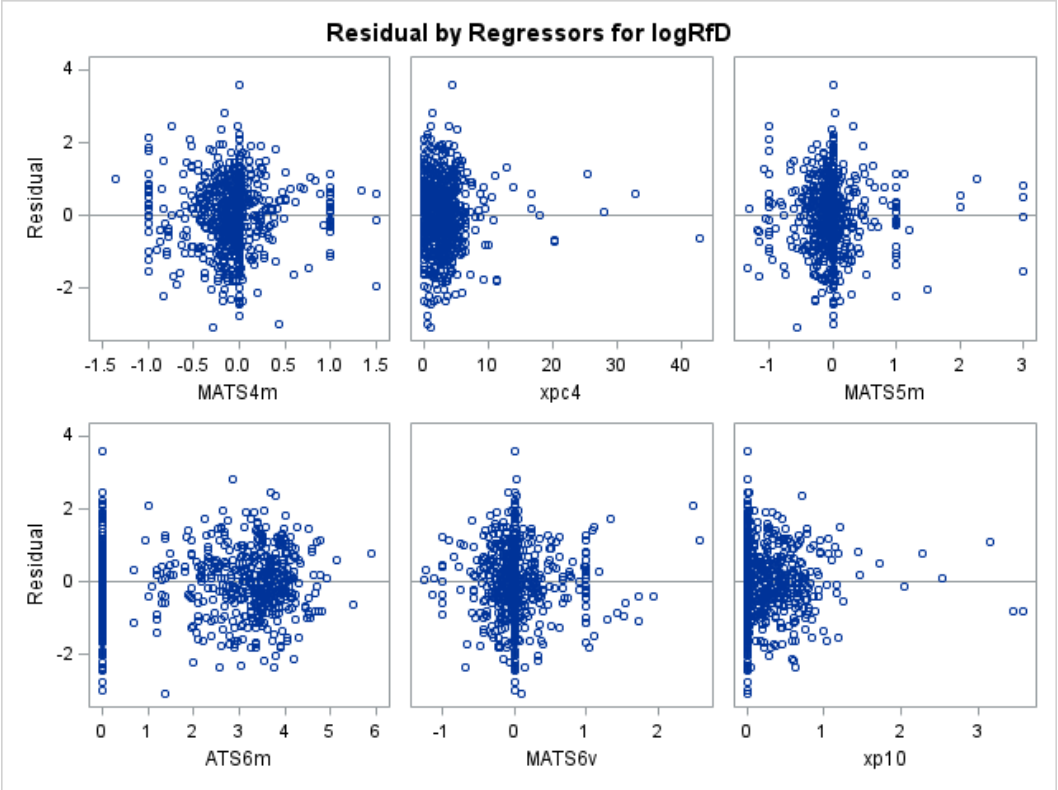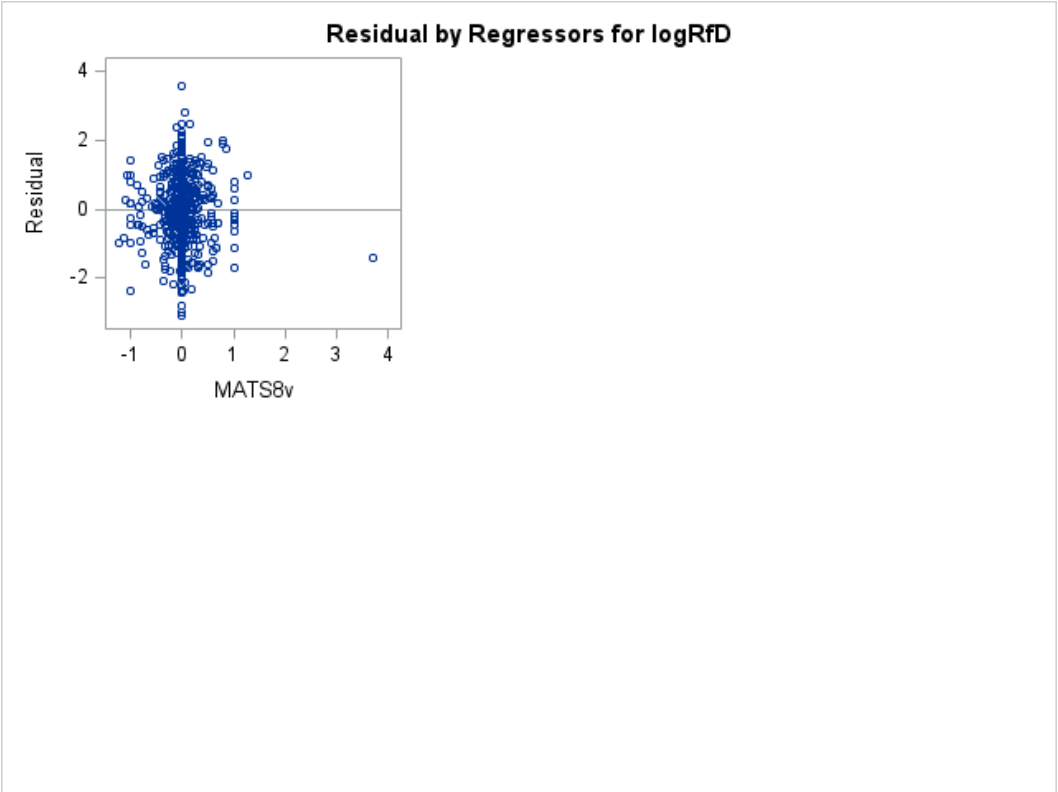

Supplement: Supplementary file 1 [file toxics-14-00529-s001.zip › Supplemental_Graph_Set_t4e07s07p30.pdf]

The REG Procedure

Model: t5e07s07p30

Dependent Variable: logRfD

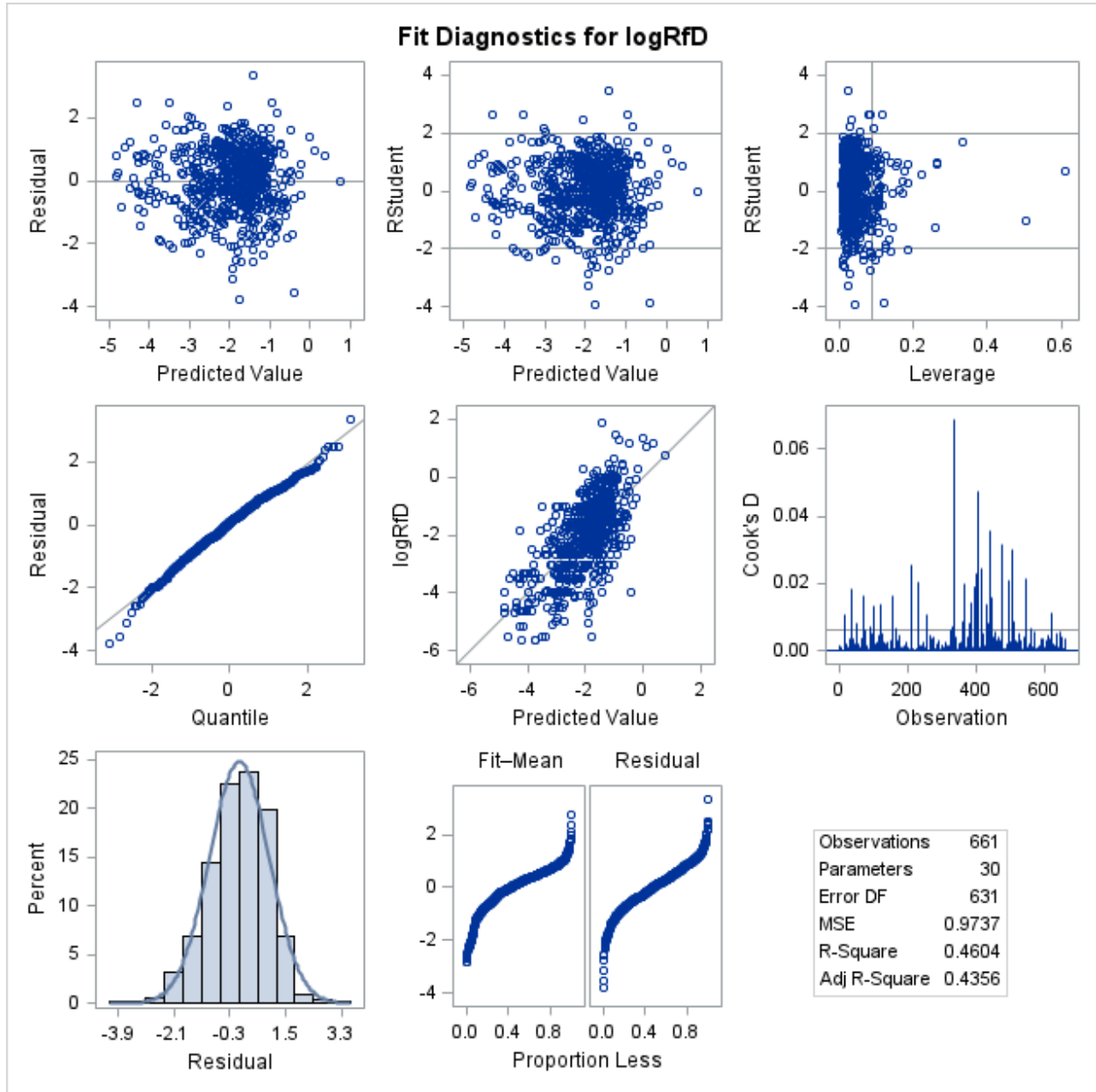

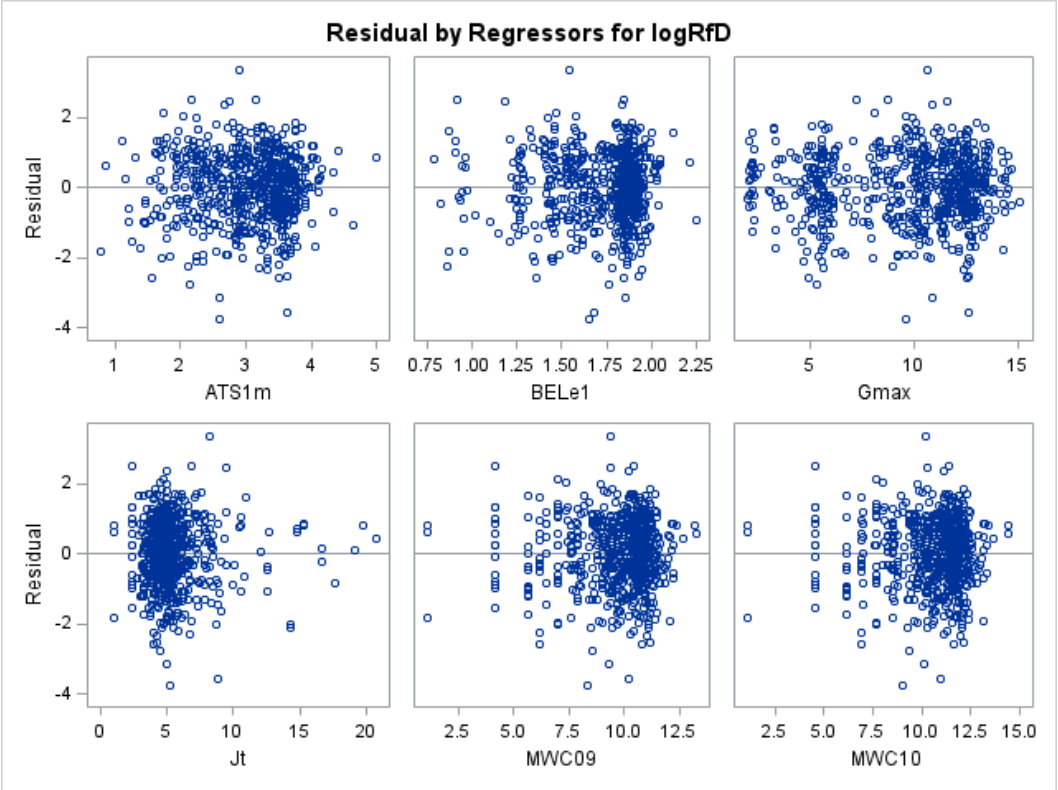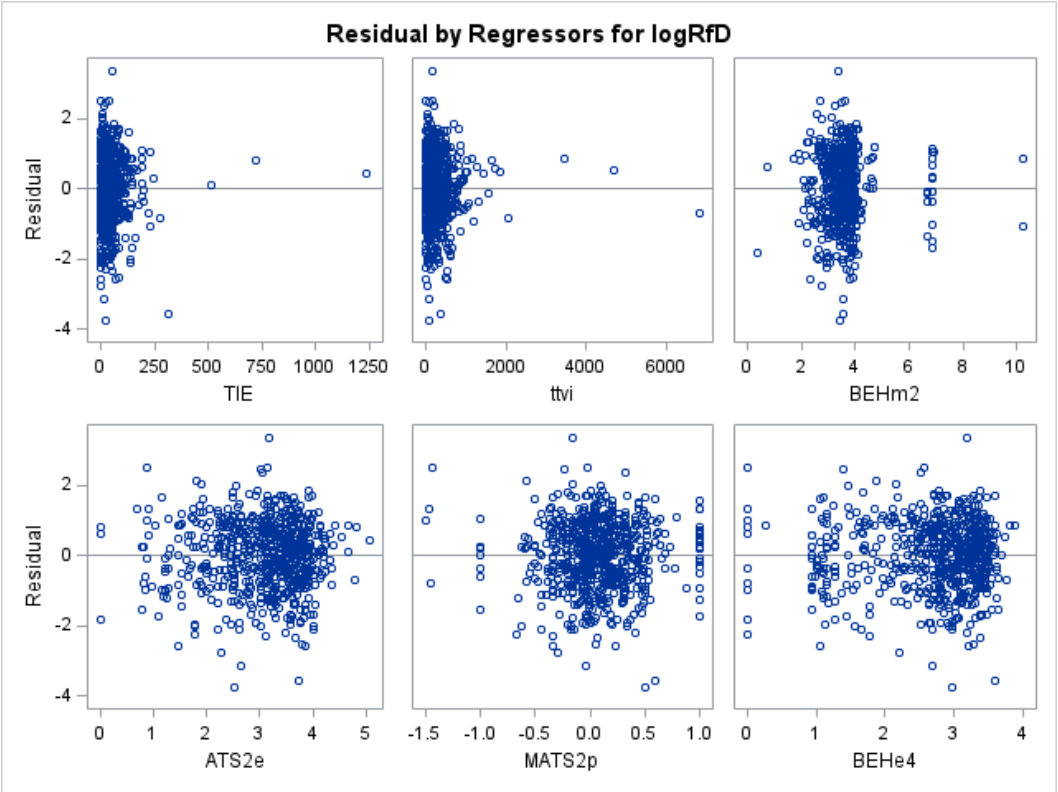

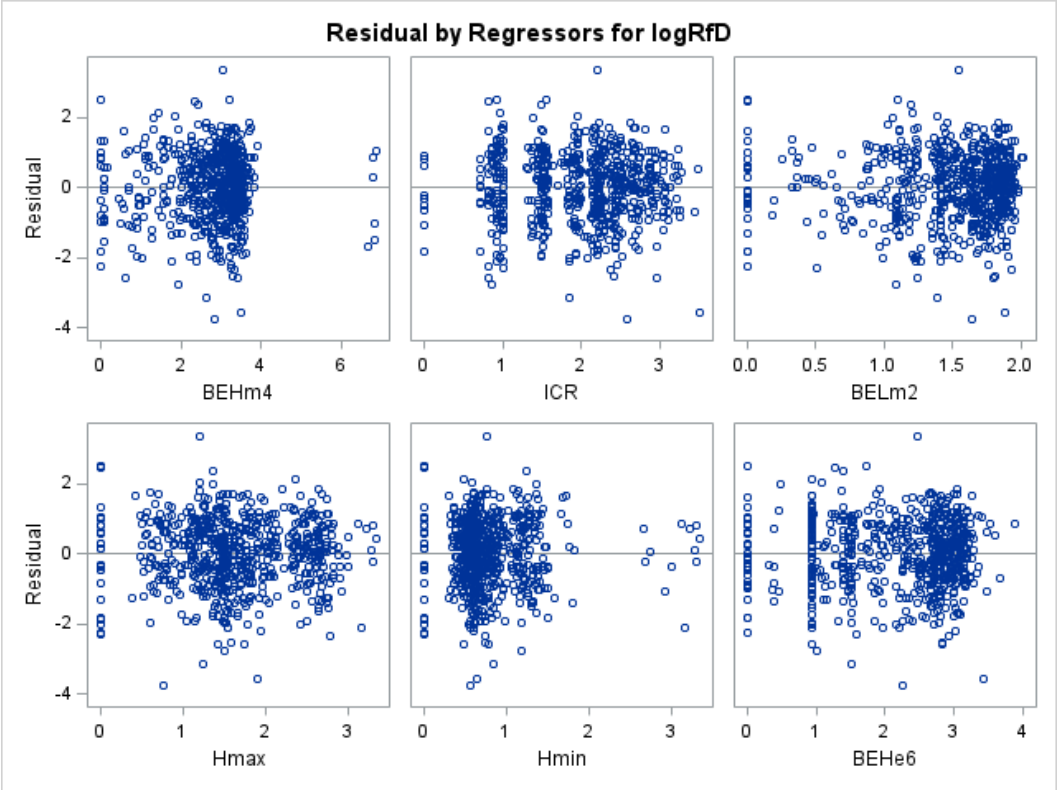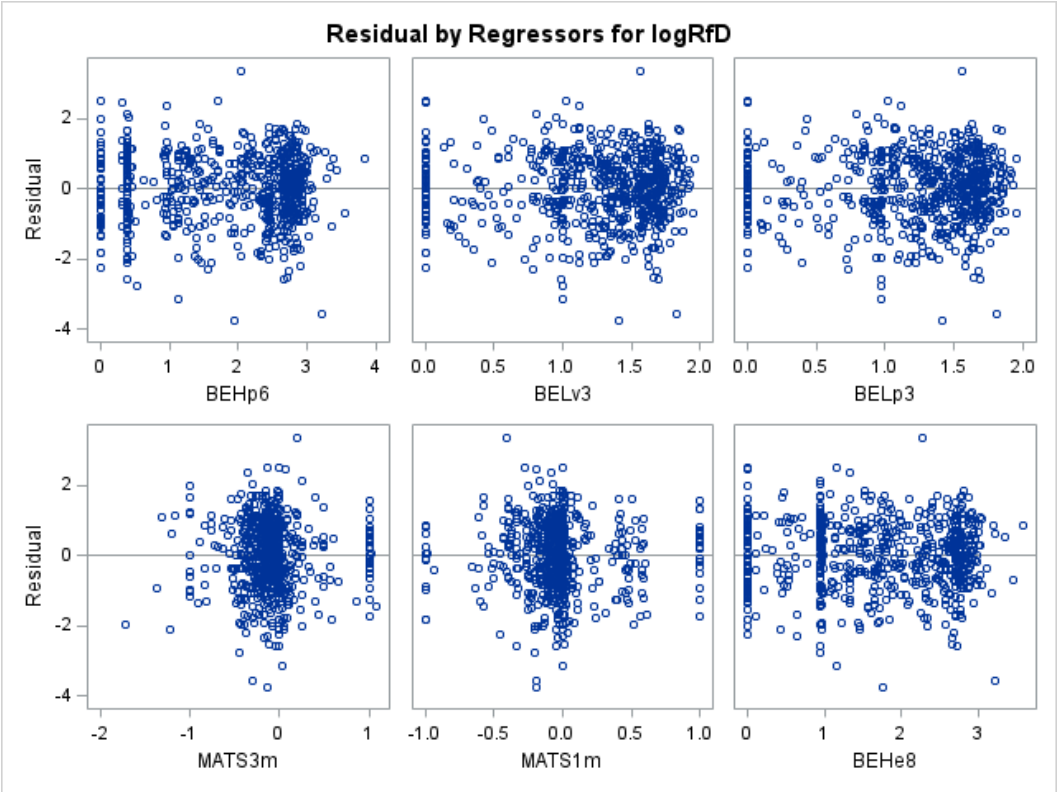

Residual by Regressors for logRfD

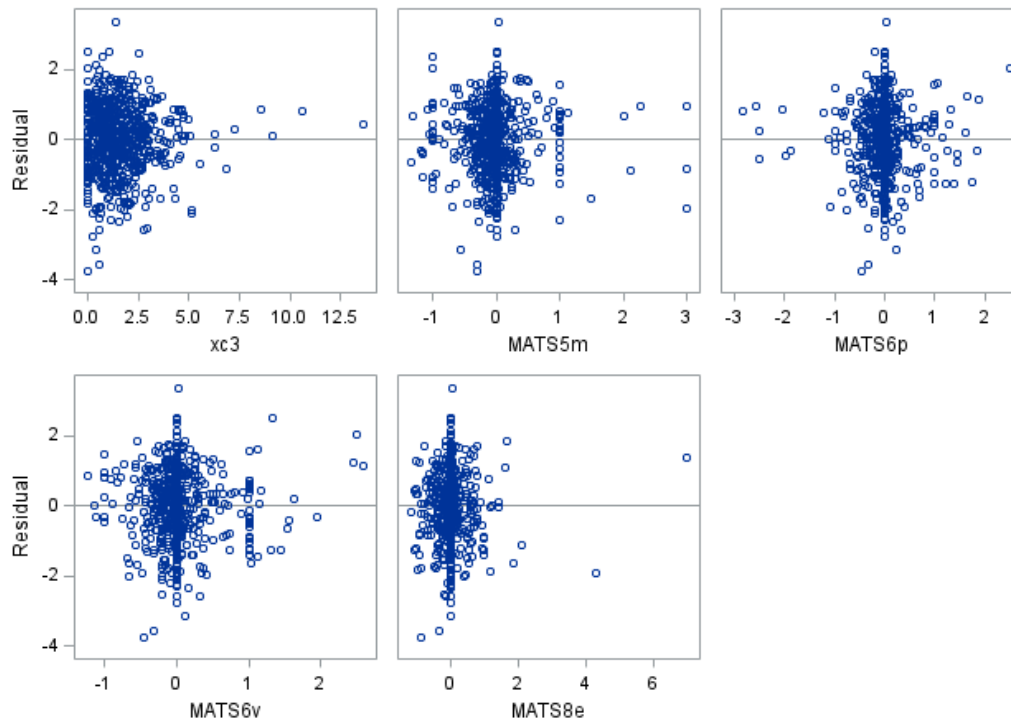

Supplement: Supplementary file 1 [file toxics-14-00529-s001.zip › Supplemental_Graph_Set_t5e07s07p30.pdf]
